# New genera and species of Neotropical Exosternini (Coleoptera, Histeridae)

**DOI:** 10.3897/zookeys.381.6772

**Published:** 2014-02-18

**Authors:** Michael S. Caterino, Alexey K. Tishechkin

**Affiliations:** 1 Department of Invertebrate Zoology, Santa Barbara Museum of Natural History, 2559 Puesta del Sol, Santa Barbara, CA 93105 USA; 2 Louisiana State Arthropod Museum, Department of Entomology, Louisiana State University, 404 Life Sciences Building, Baton Rouge, LA 70803 USA

**Keywords:** Histeridae, Histerinae, Exosternini, predator, Neotropical region

## Abstract

We describe the following 8 new genera and 23 new species of Neotropical Exosternini. *Conocassis* **gen. n.** (*Conocassis minor* **sp. n.** [type species], *Conocassis dromedaria* **sp. n.**, *Conocassis trisulcata* **sp. n.**, and *Conocassis invaginata* **sp. n.**), *Enkyosoma* **gen. n.** (*Enkyosoma rockwelli* **sp. n.**), *Pluricosta* **gen. n.** (*Pluricosta onthophiloides* **sp. n.**), *Pyxister* **gen. n.** (*Pyxister devorator* **sp. n.** [type species] and *Pyxister labralis* **sp. n.**), *Chapischema* **gen. n.** (*Chapischema doppelganger* **sp. n.**), *Scaptorus* **gen. n.** (*Scaptorus pyramus* **sp. n.**), *Lacrimorpha* **gen. n.** (*Lacrimorpha glabra* **sp. n.** [type species], *Lacrimorpha balbina* **sp. n.**, *Lacrimorpha subdepressa* **sp. n.**, and *Lacrimorpha acuminata* **sp. n.**), *Crenulister* **gen. n.** (*Crenulister grossus* **sp. n.** [type species], *Crenulister explanatus* **sp. n.**, *Crenulister dentatus* **sp. n.**, *Crenulister impar* **sp. n.**, *Crenulister umbrosus* **sp. n.**, *Crenulister simplex* **sp. n.**, *Crenulister paucitans* **sp. n.**, *Crenulister spinipes* **sp. n.**, and *Crenulister seriatus* **sp. n.**) These all represent highly distinctive and phylogenetically isolated forms, almost invariably known from very few specimens. All but one species have been collected only by passive flight intercept traps, and nothing significant is known about the biology of any of them.

## Introduction

In the course of revising all known New World Exosternini, hundreds of new species have been discovered. Many of these new species have been more or less readily assignable to known genera (including *Operclipygus* Marseul: Caterino and Tishechkin 2013; *Kaszabister* Mazur: Dégallier et al. 2012; *Mecistostethus* Marseul: Caterino et al. 2012; *Baconia*: Caterino and Tishechkin 2013b; *Hypobletus* Schmidt: Tishechkin and Caterino in prep.; *Yarmister* Wenzel: Tishechkin and Caterino in prep.). However, many have not. Phylogenetic analyses of all species of New World Exosternini, and representatives of nearly all World genera utilizing diverse morphological and molecular characters have attempted to place these numerous enigmatic species, succeeding in many cases but failing in others (Caterino and Tishechkin in review). Here we describe a number of the most distinctive and phylogenetically isolated lineages in this group, recognizing eight new genera, together containing 23 species, all of them previously undescribed.

All but one of these new species have been collected exclusively through the use of flight interception traps. While this this type of trapping is extremely valuable in biodiversity surveys, it unfortunately provides little information about the natural history of these exceptionally attractive and unusual species. We can only hope that by calling attention to their existence that our colleagues may help us to scour appropriate microhabitats to uncover the natural histories of these species.

## Materials and methods

Conventional imaging was done using a Visionary Digital's, ‘Passport’ portable imaging system, which incorporates a Canon 7D with MP-E 65 mm 1-5× macro zoom lens. Images were stacked using Helicon Focus software. SEM imaging was done on a Zeiss EVO 40 scope. Most specimens were sputter coated with gold but some uncoated specimens were examined in ‘variable pressure’ mode. We present only selected images as necessary to identify the species in this paper. However, multiple photographs of all species have been archived in MorphBank (www.morphbank.net), and are also available through the Encyclopedia of Life (www.eol.org). Following histerid conventions, total body length is measured from the anterior margin of the pronotum to the posterior margin of the elytra (to exclude preservation variability in head and pygidial extension), while width is taken at the widest point, generally near the elytral humeri.

Much of the morphological terminology used is based on Wenzel and Dybas (1941), but modified to follow more recent treatments by Helava et al. (1985), Ôhara (1994), Kanaar (1997) and Lawrence et al. (2011). We have presented an extensive discussion of Exosternini-specific morphological terminology in Caterino and Tishechkin (2013a) and refer the reader to the labeled illustrations there.

We present extensive descriptions for the majority of species. At the same time, we term most of these ‘diagnostic descriptions’, to emphasize the fact that they focus on those character systems in which differences among species are typically found. They are not intended to be exhaustive descriptions of each species’ morphology. We have attempted to make most descriptions consistent in character content and order, facilitating comparison as well as their reuse in other contexts, such as in species pages and other media, which we encourage. The ‘remarks’ sections highlight the few most important key characters of each species.

Material examined lists provide verbatim data only for holotypes and summary data for all other material, whether paratypes or non-types. Within verbatim records, data are enclosed in double quotes, with data on separate labels separated by a slash ‘/’. The summary data generally avoids excessive repetition: each record begins with the number of specimens exhibiting identical data; records separated by commas are largely identical, differing only in the datum presented, most frequently distinct dates or collectors; distinct localities are separated by semicolons, and records from distinct countries are separated by periods (full-stops).

### Collection abbreviations

CEMT Coleção de Entomologia, Universidade Federal do Mato Grosso, Cuiabá, Brazil

CHND Nicolas Degallier Collection, Paris

CMNC Canadian Museum of Nature, Ottawa, Canada

FMNH Field Museum, Chicago, USA

LSAM Louisiana State Arthropod Museum, Baton Rouge, USA

MNHN Museum National d’Histoire Naturelle, Paris, France

MSCC Michael Caterino Collection, USA

NZCS National Zoological Collection of Suriname, Paramaribo, Suriname

SEMC Snow Entomology Museum, University of Kansas, Lawrence, USA

UFPR Universidade Federal do Paraná, Curitiba, Brazil

USNM National Museum of Natural History, Washington, USA

## Taxonomy

### Conocassis gen. n.

http://zoobank.org/8B5DA462-CDB0-4DE2-AE74-82D21121AD5D

http://species-id.net/wiki/Conocassis

#### Type species.

*Conocassis minor* sp. n.

#### Description.

***Size range*:** Length 1.7–2.0 mm; width 1.2–1.5 mm; ***Body*:** rufescent to rufobrunneus, somewhat narrowly elongate, widest at humeri, abruptly narrowed anteriorly at pronotal middle, with exaggerated sculpturing throughout. ***Head*:** head deflexed relative to anterior pronotal margin; frons flat, sides weakly rounded, longitudinally strigose, with fine setigerous punctures between strigae, setae minute; frontal stria complete along margin of eye and across front, prominent, descending onto epistoma anteriorly, subangulate at middle; supraorbital stria absent; epistoma narrowed to front, apex rather narrowly emarginate, with lateral striae meeting frontal stria, convergent, nearly meeting anterad; labrum small, about twice as wide as long, apex weakly emarginate; mandibles with incisor edges evenly curved to apex, basal teeth inconspicuous; submentum broadly triangular, weakly produced into base of oral cavity, sparsely setose; mentum subtrapezoidal, apex weakly sinuate; labial palpifers prominent; labial palps 3-segmented, with basal palpomere very short, apical palpomere widest near base, subacute; maxillary cardines short, semicircular, glabrous, stipes with two setae along lateral margin; maxillary palpi 4-segmented with basal palpomere very short, palpomeres 2 and 3 similar in length and breadth, ultimate palpomere about twice as long as penultimate, widest near base, narrowed apically; antennal scape stout, anterior surface becoming longitudinally carinate in apical half, with few apical setae; funicle widening slightly to short, disclike 8^th^ antennomere; antennal club about 2.5× as long as wide, densely setose, with indistinct, denser subapical setose sensory patches on dorsal and ventral surfaces. ***Pronotum*:** widest near base, sides sinuate, strongly narrowed anterad midpoint, basal margin uneven; lateral marginal pronotal stria complete around lateral and anterior margins, though strongly sinuate at sides, submarginal stria present along sides, not parallel to marginal, joining it near anterior corner; pronotal disk with prominent dorsal process arising from entire anterior margin, narrowing and arcing more or less evenly to middle of posterior margin, sides of process longitudinally creased to deeply invaginated, dorsal surface of process coarsely reticulostrigose, sides more or less smooth; pronotal gland openings, if present, obscured by sculpturing, possibly incorporated into lateral invaginations of pronotal process. ***Elytra*:** elytron with striation generally carinate and exaggerated; epipleuron with complete marginal stria and additional stria along upper edge, paralleling outer subhumeral stria, continuing around elytral apex, variably meeting apices of dorsal striae; outer subhumeral, inner subhumeral, and dorsal striae 1-4 all complete, increasingly more strongly impressed toward suture, apices meeting apical marginal elytral suture; elytral intervals smooth to strongly microsculptured. ***Prosternum*:** prosternal keel rather narrow, base weakly produced, carinal striae complete, subparallel, united basally, meeting presternal suture anteriorly, which varies from indistinct to deeply impressed; lateral striae diverging to sides, delimiting anterior leg depression; prosternal lobe extremely reduced, no longer at midline than at sides, marginal stria obsolete. ***Mesoventrite*:** mesoventrite short, shallowly emarginate at middle, with complete marginal stria; mesometaventral stria paralleling or diverging anterad from mesometaventral suture at middle. ***Metaventrite*:** postmesocoxal and lateral metaventral striae parallel, arching toward metacoxa then anterad to metepisternum; metaventral disk weakly depressed at middle. ***Abdomen*:** 1^st^ abdominal ventrite with anterior marginal stria continued to posterior margin by lateral striae, disk rather simply and finely punctate; propygidium slightly to distinctly wider than long, rather strongly convex, sparsely to densely reticulostrigose; propygidium apparently with single pair of gland openings very close to anterior corners (obscured by sculpturing in most species); pygidium longer than basal width, sculptured as propygidium, generally smoother apicomedially. ***Legs*:** each trochanter with single seta; profemur subparallel-sided to expanded at middle of anterior margin, with anterior marginal stria delimiting microsculptured marginal area; protibiae widened from base, sides subparallel to slightly narrowing, bearing 3–5 marginal setae in apical half; protarsal groove very weakly developed; meso- and metafemora rather large, produced beyond epipleurae in repose, broad, variously widened along posterior margins; meso- and metatibiae long, widened apically, bearing 3–4 longitudinal striae on anterior surfaces; mesotibia only bearing 2–4 characteristic long, thin subapical setae, at least one of which is inserted on the posterior surface; all tarsi laterally compressed, bearing simple ventral setae, with relatively large weakly curved claws. ***Male genitalia*** (Fig. 4): Paired accessory sclerites present; T8 with broad basal and narrower apical emarginations, line of basal membrane attachment complete, just distad basal emargination, ventral apodemes well developed, nearly meeting along midline; S8 with halves separated, apical guides moderately and evenly developed from base to apex, each apex with single prominent seta; 9^th^ tergite with very weak ventrolateral apodemes, apices narrow, subacute; T10 completely divided; S9 broad at base, narrowest near apex, head broad, subquadrate apically, with small apical emargination, more strongly sclerotized along midline; tegmen broad basally, strongly narrowed to apex, narrowly divided apically, median foramen basad apical narrowing, moderately to strongly curved ventrad in apical half; basal piece about one-third tegmen length; median lobe short, simple, from one-fourth to one-third tegmen length. ***Female genitalia*:** T8 forming a single plate, apically emarginate; S8 tripartite, basal baculi convergent proximally; S9 elongate, articulated with strap-shaped extension from apex of S8; T10 entire; overall ovipositor rather short; valvifers paddle-shaped, paddles nearly one-half total length; coxites strong, slightly longer than broad, two-thirds length of valvifers, strongly bidentate, with strengthening ridge on inner face; gonostyle present; bursa copulatrix membraneous, weakly expanded; spermatheca gradually expanded, apically bulbous, with slightly expanded spermathecal gland attached near its base.

#### Diagnosis.

**Figure 1.**
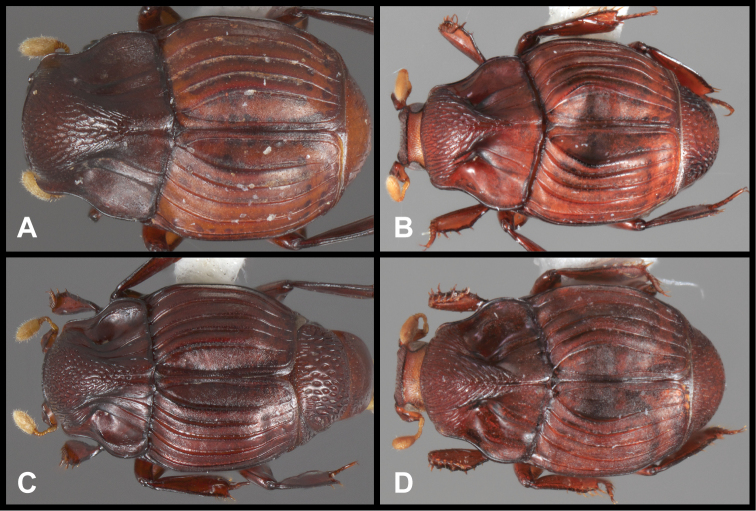
*Conocassis* spp. dorsal habitus. **A** *Conocassis minor* **B** *Conocassis dromedaria* **C** *Conocassis trisulcata* **D** *Conocassis invaginata*.

**Figure 2.**
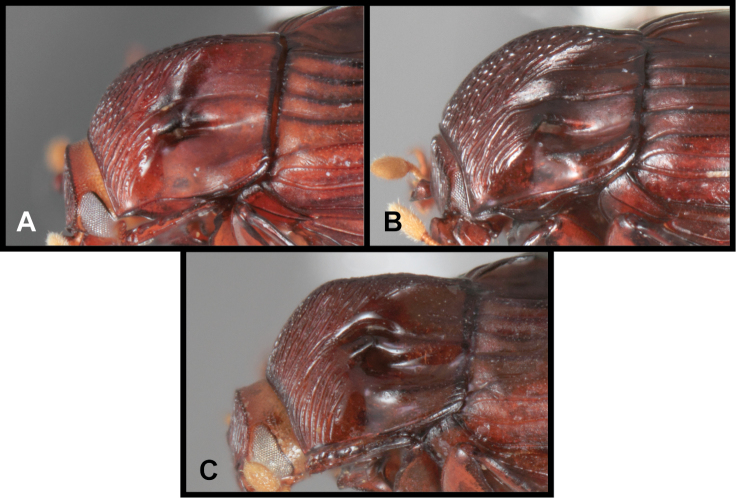
*Conocassis* spp., lateral view of pronotum. **A** *Conocassis dromedaria* **B** *Conocassis trisulcata* **C** *Conocassis invaginata*.

**Figure 3.**
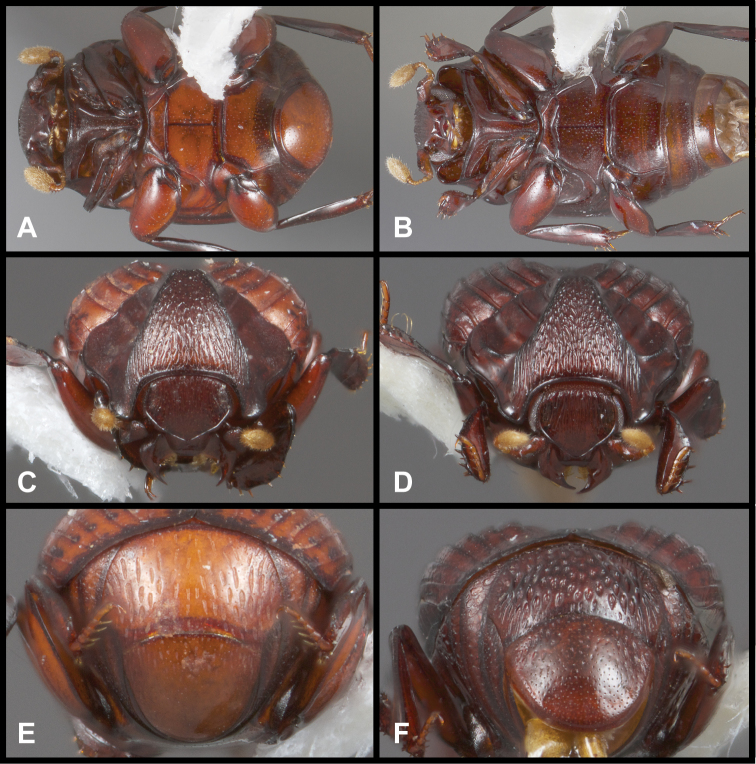
*Conocassis* spp. **A** *Conocassis minor*, ventral view **B** *Conocassis trisulcata*, ventral view **C** *Conocassis minor*, anterior view **D** *Conocassis trisulcata*, anterior view **E** *Conocassis minor*, posterior view of pygidia **F** *Conocassis trisulcata*, posterior view of pygidia.

This highly distinctive genus scarcely needs a diagnosis. Its prominent pronotal process (Figs 1, 2) is unique and unmistakable. Its assignment perhaps to tribe could be problematic, as it exhibits no hint of an emargination of the prosternal keel (Fig. 3A–B). However, both male and female genitalia, as well as DNA sequences place it unambiguously as deeply nested within the neotropical Exosternini.

**Figure 4.**
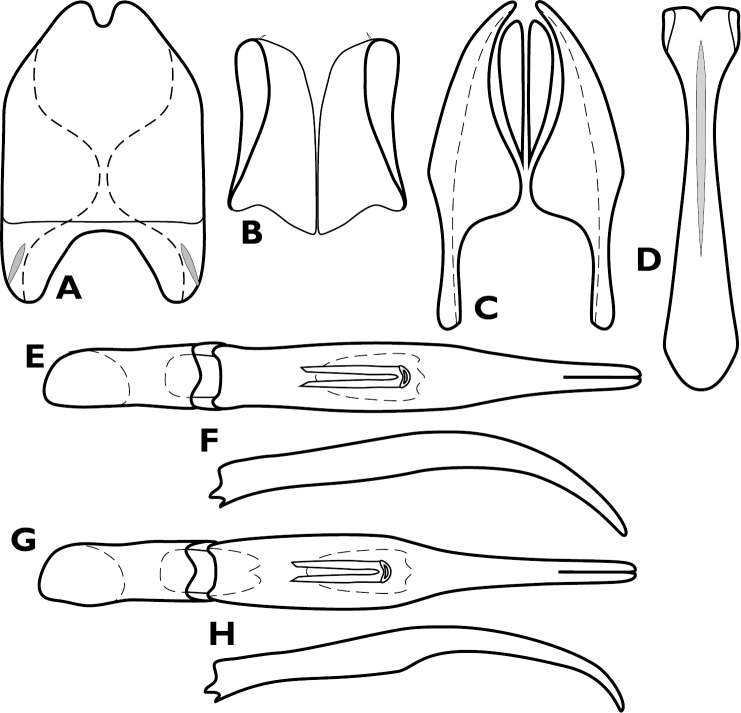
*Conocassis* spp., male genitalia. **A-F** *Conocassis minor* **A** 8^th^ tergite **B** 8^th^ sternite **C** 9^th^ and 10^th^ tergites **D** 9^th^ sternite **E** Aedeagus, dorsal view **F** Aedeagus, lateral view **G–H** *Conocassis dromedaria* **G** Aedeagus, dorsal view **H** Aedeagus, lateral view.

#### Remarks.

Phylogenetic analyses to date place *Conocassis* as the sister group of *Kaszabister*, a group of 4 species which are inquilines of fire ants (*Solenopsis* spp.) (Caterino and Tishechkin in review). There are few obvious similarities between these apart from generally exaggerated surface sculpturing (to a much lesser degree in *Kaszabister*). We were very fortunate to have collected a DNA quality specimen of *Conocassis minor* during our own fieldwork, its sequence providing some confidence in its general placement. *Kaszabister*, on the other hand, has not yet been sequenced, so a more rigorous test of their close relationship remains to be carried out.

#### Etymology.

The genus name means ‘conical helmet’ referring to the anterior process of the pronotum. The gender of the name is feminine.

#### Key to species of *Conocassis*

**Table:** 

1	Sides of pronotal shield with at least one deep invagination (Fig. 2A–C); mesometaventral stria narrowly arched anterad at middle, departing from mesometaventral suture (Fig. 3B); larger, darker species	2
–	Sculpturing of sides of pronotal shield more superficial (Fig. 1A); mesometaventral stria barely departing anteriorly from mesometaventral suture (Fig. 3A); smaller, paler species	*Conocassis minor*
2	Basal half of 4^th^ elytral stria strongly bulged outward, interval deeply excavate medially (Fig. 1B, D)	3
–	Basal half of 4^th^ elytral stria more or less evenly arcuate from base to apex, interval not so deeply excavate medially (Fig. 1C)	*Conocassis trisulcata*
3	Pronotal shield in lateral profile evenly curved from anterior to posterior margins (Fig. 2B)	*Conocassis dromedaria*
–	Pronotal shield in lateral profile straight in basal half, abruptly curving downward from near midpoint to apical margin (Fig. 2C)	*Conocassis invaginata*

### Conocassis minor sp. n.

http://zoobank.org/27475A8C-6617-4B8C-BABF-C099F0CF575D

http://species-id.net/wiki/Conocassis_minor

**Map 1.**
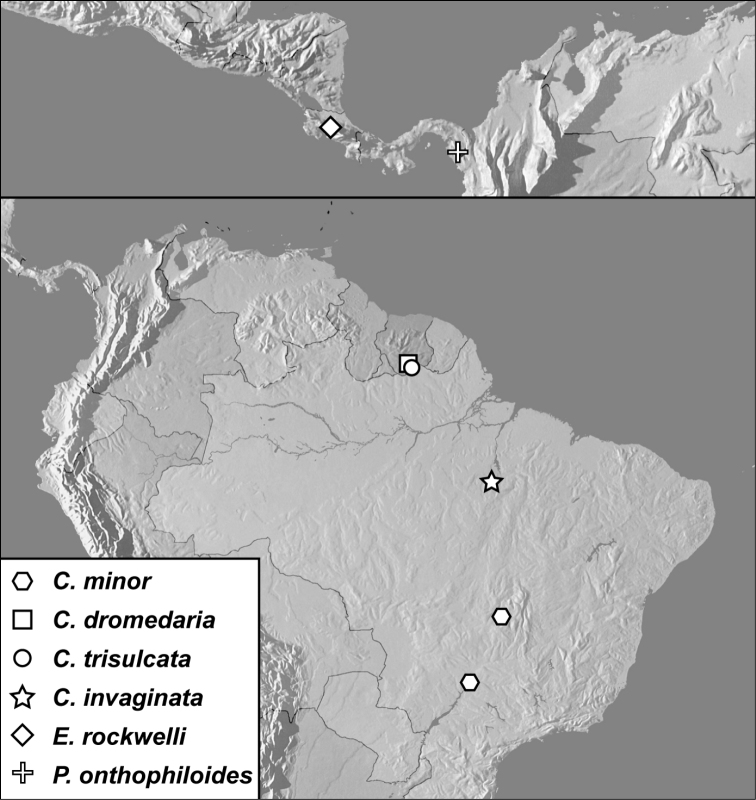
Specimen records of *Conocassis* spp., *Enkyosoma rockwelli*, and *Pluricosta onthophiloides*.

Figs 1A , 3A, C, E , 4A–F , Map 1

#### Type locality.

BRAZIL: Distrito Federal, Reserve IBGE [15.95°S, 47.88°W].

#### Type material.

**Holotype male:** “**BRASIL:** **Dist. Federal**, Brasilia, Res. Ecol. de IBGE, 15°5.5'S, 47°53'W, Lin. 1, Pto. 3. Armad. janela, area queimada. 20.i.1998” / “Caterino/Tishechkin Exosternini Voucher EXO-00099” (UFPR). **Paratypes** (2): same data as type (CHND, FMNH).

Other material: **BRAZIL:** **Mato Grosso do Sul**, cerradão fragment nr. Selviria, 20.3354°S, 51.4095°W, flight intercept trap [FIT], 30.xi-3.xii.2011, M.S. Caterino & A.K. Tishechkin, DNA extraction #MSC-2273, voucher EXO-00932 (MSCC).

#### Diagnostic description.

Length: 1.6–1.7 mm, width: 1.1–1.2 mm; as in generic description, with the following specific characters: body rufescent; frontal stria extending only to base of epistoma; apical margin of epistoma deeply emarginate; side of pronotal process with two longitudinal creases, neither deeply invaginated; pronotal disk lacking basolateral carina; pronotal process more or less evenly rounded in lateral profile; pronotal disk very finely alutaceous behind median process; all elytral striae rather weakly carinate; median elytral interval shallowly depressed in basal half, texture of median interval finely granular throughout; no trace of 5^th^ dorsal stria present in median interval; texture of outer intervals finely granulate basally, smoother apically; epipleural margin granulate but not strigose; presternal suture not impressed across middle, carinal striae only vaguely extended to sides; protibiae slightly narrowed in apical half, apex rounded; protibial spurs inconspicuous; abdominal ventrites 2-4 faintly and shallowly punctate at sides; propygidium moderately convex, slightly shorter than pygidium along midline; propygidial punctures elongate, only moderately deep, more so at sides, punctures faintly alutaceous within; pygidium finely strigose in basal third, finely punctate apically; male genitalia (Fig. 4A–F) as for generic description except tegmen widest just distad midpoint, ventral curvature rather even in apical half.

#### Remarks.

This species is easily distinguished from the other three species in this genus. It is distinctly smaller and paler in color, and generally has the dorsal sculpturing less exaggerated (Fig. 1A). The elytral striae are rather simply carinate, and the lateral creases on the sides of the pronotal process are not at all invaginated as they are in the following three species.

#### Etymology.

This species name refers to the fact that it is the smallest known member of the genus.

### Conocassis dromedaria sp. n.

http://zoobank.org/95C4F58A-408D-4946-B12D-B943DF5136C7

http://species-id.net/wiki/Conocassis_dromedaria

Figs 1B , 2A , 4G–H , Map 1

#### Type locality.

SURINAME: Sipaliwini, upper Palumeu River [2.4770°N, 55.6294°W].

#### Type material.

**Holotype male:** “**SURINAME:** **Sipaliwini**, CI-RAP Survey camp 1, upper Palumeu, 225m, 2.47700°N, 55.62941°W, Flight intercept. 10–16.iii.2012, A.E.Z. Short, SR12-0310-TN1” / “Caterino/Tishechkin Exosternini Voucher EXO-03047” (NZCS).

#### Diagnostic description.

Length: 1.9 mm, width: 1.4 mm; as for generic description, with the following specific characters: body rufobrunneus; frontal stria extending only onto base of epistoma; side of pronotal process with two distinct creases, the lowermost narrowly, deeply invaginated; pronotal disk lacking carina extending anterad from basolateral corner; pronotal process rather evenly rounded from base to apex in lateral profile; pronotal disk very finely alutaceous behind median process; median elytral interval strongly depressed in basal half, the 4^th^ dorsal stria strongly elevated and displaced laterad, texture of median interval finely granular throughout; texture of outer intervals finely alutaceous basally, becoming smooth posteriorly; epipleural margin granulate but not strigose; no trace of 5^th^ dorsal stria present; presternal suture deeply impressed, especially at middle; abdominal ventrites 2-4 with sparse, oblique strigae at sides; propygidium strongly convex, about as long as pygidium along midline; propygidial punctures very elongate, coarse and deep, especially at sides, alutaceous within; pygidium strigose in basolateral corners, becoming simply, finely punctate apicomedially; male genitalia (Fig. 4G–H) as for generic description except tegmen widest just basad midpoint, apices long and narrow, ventral curvature moderate to near apex where it is abruptly bent ventrad.

#### Remarks.

This species and the following two are very similar, closely related, and difficult to separate. All are larger, darker, and more strongly sculptured than *Conocassis minor*, and can easily be separated from it. However, differences among them are more subtle. *Conocassis dromedaria* and *Conocassis invaginata* appear most similar, with the strong basal depression in the median elytral interval (Fig. 1B, D), and the 4^th^ stria very strongly elevated and displaced laterad. These two can be separated by the narrowly open lower pronotal invagination, and poorly developed upper pronotal invagination of *Conocassis dromedaria* (Fig. 2A). This species also lacks a basolateral carina on the pronotal disk that the other two share. The more completely granulate elytral intervals of *Conocassis invaginata* (Fig. 1D) also set it apart from both the others, in which the intervals are distinctly smoother apically. With *Conocassis dromedaria* and *Conocassis trisulcata* represented only by single specimens, and only *Conocassis dromedaria* represented by a male, their status will have to be reassessed later in light of more material.

#### Etymology.

This species is named for the camel-like hump on the pronotum, from the specific name of the one-humped dromedary.

### Conocassis trisulcata sp. n.

http://zoobank.org/0AB37D8D-A738-4067-BB58-855BFEC48639

http://species-id.net/wiki/Conocassis_trisulcata

Figs 1C , 2B , 3B, D, F , Map 1

#### Type locality.

SURINAME: Sipaliwini, upper Palumeu River [2.4770°N, 55.6294°W].

#### Type material.

**Holotype female:** “**SURINAME:** **Sipaliwini**, CI-RAP Survey camp 1, upper Palumeu, 225m, 2.47700°N, 55.62941°W, Flight intercept. 10-16.iii.2012, A.E.Z. Short, SR12-0310-TN1” / “Caterino/Tishechkin Exosternini Voucher EXO-02504” (NZCS).

#### Diagnostic description.

Length: 2.0 mm, width: 1.5 mm; as for generic description, with the following specific characters: body rufobrunneus; frontal stria extending to middle of epistoma; side of pronotal process with three distinct creases, only the lowermost narrowly, deeply invaginated; pronotal disk with distinct carina extending anterad from basolateral corner toward median invagination; pronotal process rather evenly rounded from base to apex in lateral profile; pronotal disk very finely alutaceous behind median process; median elytral interval only moderately depressed in basal half, the 4^th^ dorsal stria not displaced laterad, only weakly arcuate throughout length, texture of median interval finely granular throughout; texture of outer intervals finely alutaceous basally, becoming smooth posteriorly; epipleuron vertically strigose on and above marginal bead; no trace of 5^th^ dorsal stria present; presternal suture marked by anteriorly divergent prosternal carinal striae at sides, not impressed across middle; abdominal ventrites 2-4 only very faintly strigose at sides; propygidium strongly convex, about as long as pygidium along midline; propygidial punctures coarse and deep, slightly elongate, only slightly more so at sides, punctures largely smooth within; pygidium strigose in basolateral corners, becoming simply, finely punctate apicomedially.

#### Remarks.

In addition to the characters remarked under the preceding species, this species’ relatively shallow median elytral depression (Fig. 1C), and simply carinate and arcuate 4^th^ dorsal stria will separate it, as will the presence of three distinct lateral creases on the pronotal process (Fig. 2B), only the lowermost of which is distinctly invaginated. This species also lacks an indication of the presternal suture at the middle (Fig. 3B).

#### Etymology.

This species is named for the three distinct lateral pronotal creases.

### Conocassis invaginata sp. n.

http://zoobank.org/7987E394-93FB-4AA5-BCF5-BBD6707EDD1B

http://species-id.net/wiki/Conocassis_invaginata

Figs 1D , 2C , Map 1

#### Type locality.

BRAZIL: Pará, Carajas [6.06°S, 50.2°W].

#### Type material.

**Holotype female:** “Octobre 1984, piége d’interception CARAJAS, PARÁ, N. DEGALLIER” / “Caterino/Tishechkin Exosternini Voucher EXO-00008” (UFPR). **Paratype female:** **BRAZIL:** **Pará**, Carajas, S. Norte, xi.1984, N. Degallier (CHND).

#### Diagnostic description.

Length: 1.9 mm, width: 1.4 mm; as for generic description, with the following specific characters: body rufobrunneus; frontal stria extending to near apex of epistoma; side of pronotal process with two distinct creases, the lowermost deeply invaginated, more broadly open, the second, more dorsal crease also rather deeply invaginated; pronotal disk with carina extending anterad from basolateral corner toward median invagination; pronotal process abruptly rounded to apex only from middle in lateral profile; pronotal disk very finely alutaceous behind median process; median elytral interval strongly depressed in basal half, the 4^th^ dorsal stria strongly elevated and displaced laterad, texture of median interval finely granular throughout; fragments of 5^th^ dorsal stria present in median interval; texture of outer intervals finely granulate throughout, not smoother apically; epipleural margin granulate but not strigose; presternal suture impressed across middle; abdominal ventrites 2-4 with sparse, oblique strigae at sides; propygidium strongly convex, about as long as pygidium along midline; propygidial punctures very elongate, coarse and deep, especially at sides, alutaceous within; pygidium strigose in basolateral corners, becoming simply, finely punctate apicomedially.

#### Remarks.

As noted under the preceding two species, this species is best recognized by the presence of only two lateral creases on the pronotal process (Fig. 2C), both of which are deeply invaginated. The completely granulate elytral intervals and presence of fragments of the 5^th^ dorsal stria (Fig. 1D) are also unique.

#### Etymology.

This species is named for the fact that it has the deepest pronotal invaginations in the genus.

### Enkyosoma gen. n.

http://zoobank.org/514A6CCE-FA73-4B93-8E75-6A076491AE53

http://species-id.net/wiki/Enkyosoma

#### Type species.

*Enkyosoma rockwelli* sp. n.

#### Description.

This genus differs from other Exosternini in the following combination of characters: body widest behind middle, subdepressed, somewhat flattened dorsally, metaventrite rather abruptly convex ventrally, glabrous, lacking secondary punctation, and almost entirely lacking typical striae; frons more or less coplanar with vertex; labrum broad, apically emarginate, lateral margins with conspicuous setal fringe; mandibles with short incisor edges; antennal scape elongate, slender, curving posteroventrad beneath eye; funicle about as long as scape, weakly widened distally, the 8^th^ antennomere cupuliform, no shorter than preceding antennomeres; antennal club elongate oval, completely tomentose, with two nearly complete setose annuli more distinctly interrupted on dorsal surface, only slightly curved basad at middle; eye substantially reduced; pronotum lacking prescutellar impression, though fine prescutellar fovea may be present, with three median gland openings on each side, one in anterior angles, one behind eye about one funicle width behind anterior margin, one displaced posterad on disk; three distinct gland openings also present along lateral pronotal margin; elytra with few striae extremely fine, inconspicuous; prosternal keel depressed, rather broad, produced at base; prosternal lobe deflexed; mesoventrite anteriorly emarginate; metaventrite strongly convex posteriorly, posterior margin arcuate; propygidium transverse, with single gland openings in anterolateral corners; pygidium large, apically rounded, without gland openings or marginal striae; trochanter with 2-3 short apical setae; femora moderately broad, slightly flattened; protibia subtriangular, with outer edge weakly outwardly rounded, not distinctly toothed, with numerous distinct marginal spines; two small protibial spurs present; all tarsi rather long and curving, with numerous ventral spines, those of protarsus (of male only?) strongly spatulate; mesotibia moderately expanded toward apex, metatibia less so, both with nearly complete series of marginal spines; male genitalia (Fig. 6) with paired accessory sclerites present; apices of S8 bearing only very fine, inconspicuous setae; T10 completely divided; S9 with head broad, subquadrate, with complete apical flange; tegmen narrow and elongate, sides parallel in basal half, narrowed to thin, ventrally curved apex, median foramen basad apical narrowing, ventral surface with basal tooth formed by thin median keel; Female not known.

#### Remarks.

**Figure 5.**
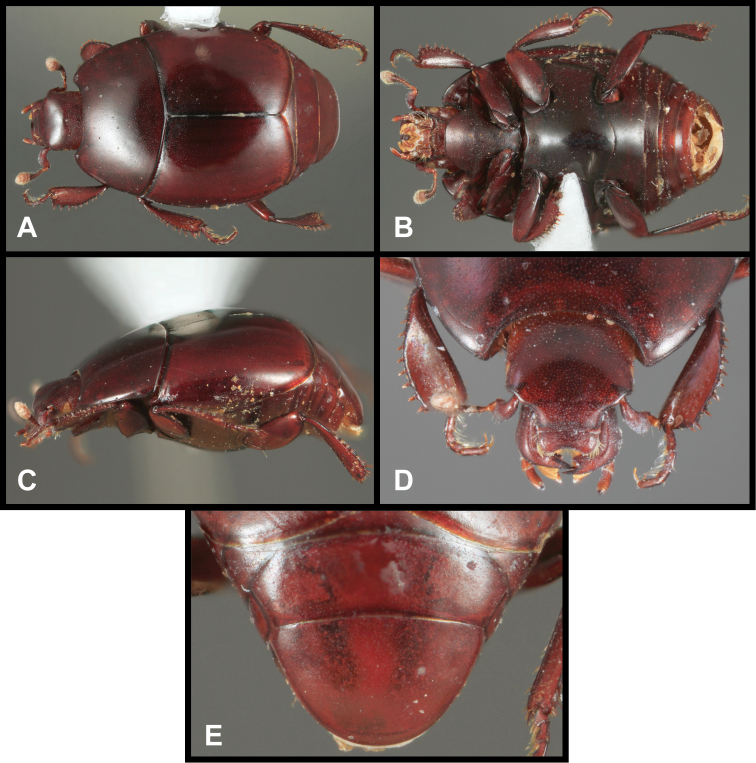
*Enkyosoma rockwelli*. **A** Dorsal habitus **B** Ventral habitus **C** Lateral habitus **D** Anterior view of head **E** Posterior view of pygidia.

This genus is easily recognized by its relatively large, rounded, subdepressed body form (Figs 5A-C), and its complete lack of elytral striae. Its spinose, slightly dilated tibiae and strongly convex metaventrite are also unique features.

In our recent analysis of Exosternini relationships (Caterino and Tishechkin in review) *Enkyosoma* is resolved as closely related to *Scaptorus* and *Chapischema*, the three of them forming the sister group to *Operclipygus*. However, only three characters change on the branch supporting this group (loss of 5^th^ dorsal elytral stria, reduction to a single seta on the protrochanter, and the differentiation of the proximal apodemes of the male median lobe), and none are particularly substantial or unique. In gross morphology there are no obvious similarities among the three.

**Figure 6.**
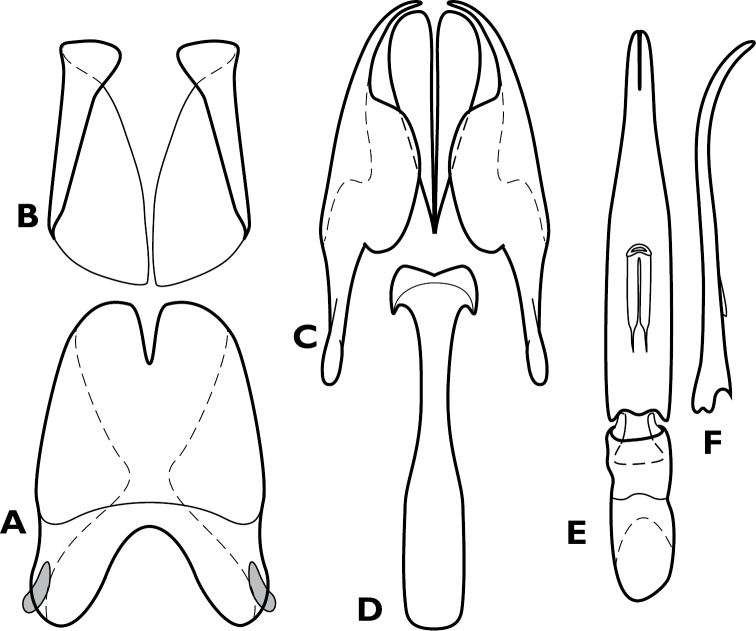
*Enkyosoma rockwelli*, male genitalia. **A** 8^th^ tergite **B** 8^th^ sternite **C** 9^th^ and 10^th^ tergites **D** 9^th^ sternite **E** Aedeagus, dorsal view **F** Aedeagus, lateral view.

#### Etymology.

From the Greek, literally ‘pregnant body’, referring to the strongly convex metaventrite.

### Enkyosoma rockwelli sp. n.

http://zoobank.org/B2A17604-CE7D-4383-8BB4-4742770CD804

http://species-id.net/wiki/Enkyosoma_rockwelli

Figs 5 , 6 , Map 1

#### Type locality.

COSTA RICA: Puntarenas, Monteverde [10.3194°N, 84.8158°W].

#### Type material.

**Holotype male:** “**COSTA RICA:** **PUNTARENAS**, Monteverde, Estacion Biologica Monteverde 10°19'10"N, 84°48'57"W, 1730m, 12.VI.2001 R.Anderson, montane forest litter, 2001-107H” / “Caterino/Tishechkin Exosternini Voucher EXO-00175” (CMNC).

#### Description.

***Size range*:** Length 3.0 mm; width 2.7 mm; ***Body*:** body elongate ovoid, widest behind middle, subdepressed, dorsally somewhat flattened, more convex ventrally, dark rufescent, glabrous, with conspicuous ground punctation throughout, lacking secondary punctation, and almost entirely lacking typical striae. ***Head*:** frons elongate, largely coplanar with vertex, weakly depressed at middle; frontal and supraorbital striae absent; epistoma wide, somewhat deflexed, weakly emarginate apically; labrum large, about one-third as long as wide, apical margin deeply emarginate, lateral margins with conspicuous setal fringe; mandibles with short incisor edges, left bearing prominent basal tooth, right mandible with weaker basal tooth; submentum wide, slightly depressed relative to surrounding genae, anterior margin weakly outwardly arcuate; mentum trapezoidal, narrowed anteriorly, apical margin more or less entire; labial palps three segmented, basal segment short, penultimate and ultimate palopmeres elongate; submentum and mentum bearing numerous elongate setae; maxillary cardo shining, glabrous, stipes microsculptured and with numerous long setae; maxillary palp four segmented, rather stout; antennal scape elongate, not markedly widened apically, curving posteroventrad beneath somewhat reduced compound eye; funicle about as long as scape, weakly widened distally, the 8^th^ antennomere cupuliform, no shorter than preceding antennomeres; antennal club elongate oval, completely tomentose, with two nearly complete setose annuli more distinctly interrupted on dorsal surface, only slightly curved basad at middle. ***Pronotum*:** pronotum weakly convex, sides faintly arcuate from base to apices; prescutellar impression absent, though fine prescutellar fovea may be present; pronotal disk with three median gland openings on each side, one in anterior angles, close to corner, one behind eye about one funicle width behind anterior margin, one directly posterad this one, just behind pronotal midpoint; three distinct gland openings also present along lateral pronotal margin; marginal stria complete along lateral and anterior margins; fragments of lateral submarginal stria visible near anterior corners. ***Elytra*:** elytra with few striae extremely fine, inconspicuous; epipleuron with single complete marginal stria and fragments of additional epipleural striae detectable, oblique humeral stria visible near base, 1^st^ dorsal stria finely impressed in apical two-thirds, 2^nd^ stria visible in basal half, slightly abbreviated from base, 3^rd^ stria present in basal third, 4^th^, 5^th^ and sutural striae entirely absent; elytral disk with very fine granular microsculpture faintly impressed toward apices. ***Prosternum*:** prosternal keel depressed, rather broad, produced at base, lacking striae; prosternal lobe about three-fourths length of keel, deflexed, lacking marginal stria; both prosternal keel and lobe rather densely microsculptured. ***Mesoventrite*:** mesoventrite broad, subquadrate, anterior margin emarginate, marginal stria faintly impressed. ***Metaventrite*:** metaventrite strongly convex posteriorly, posterior margin arcuate; mesometaventral, postcoxal and inner lateral metaventral striae absent, metaventral disk smooth at middle, more distinctly microsculptured at sides. ***Abdomen*:** abdominal ventrites 1-4 lacking striae, more or less distinctly microsculptured throughout; propygidium short, transverse, with single gland openings in anterolateral corners; pygidium large, apically rounded; propygidium and pygidium with very fine ground punctation and transverse waves of microsculpture. ***Legs*:** each trochanter with 2-3 short apical setae; femora moderately broad, slightly flattened; protibia subtriangular, with outer edge weakly outwardly rounded, not distinctly toothed, but set with about 9 distinct marginal spines; two small protibial spurs present; all tarsi rather long and curving, with numerous ventral spines, those of protarsus (of male only?) strongly spatulate; mesotibia moderately expanded toward apex, metatibia much less so, both with nearly complete series of marginal spines. ***Male genitalia*** (Fig. 6): Paired accessory sclerites present; T8 with broad basal and narrower apical emarginations, line of basal membrane attachment complete distad basal emargination, ventral apodemes well developed, narrowing ventrally, slightly separated along midline; S8 with halves approximate at base, apical guides widening from base to apex, apices narrowly rounded, bearing only very fine, inconspicuous setae; T9 with base of dorsal flaps rather protuberant above proximal apodemes, ventrolateral apodemes well developed, T9 apices narrow, convergent subacute; T10 elongate, completely divided; S9 with sides subparallel in basal half, narrowest distad midpoint, head broad, subquadrate apically, with complete apical flange, apex not emarginate; tegmen narrow and elongate, sides parallel in basal half, narrowed to thin, ventrally curved apex, median foramen basad apical narrowing, ventral surface with basal tooth formed by thin median keel; basal piece nearly one-half tegmen length; median lobe about one-third tegmen length, proximal arms thinned basally. ***Female*:** not known.

#### Remarks.

This species is only known from the type specimen.

#### Etymology.

We name this species for Mr. Marvin Rockwell, one of the original Quaker founders of the Costa Rican community of Monteverde, who has helped many visitors (the senior author included) better appreciate the biodiversity of Costa Rica.

### Pluricosta gen. n.

http://zoobank.org/521A7B18-FA66-4C18-9D6F-0B29AC444B56

http://species-id.net/wiki/Pluricosta

#### Type species.

*Pluricosta onthophiloides* sp. n.

#### Description.

This genus differs from other Exosternini in the following combination of characters: body round, strongly convex, with strong elytral ridges, rufescent, glabrous; frons and epistoma depressed along midline, frontal stria present; labrum wide, shallowly emarginate apically; mandibles with weakly arcuate incisor edges lacking basal teeth; antennal scape slightly wider near base, narrowed apically, with longitudinal carina along inner anterior edge; funicle shorter than scape, weakly widening apically, antennomere 8 short, cupuliform, not disc-like; antennal club slightly elongate oval, largely tomentose, with only indistinct subapical sensory patches; pronotum rather strongly convex, with two gland openings on each side, anterior opening simple, along anterior margin behind eye, posterior opening with secondary annulus, displaced posterad to near middle of disk; prescutellar impression absent; elytra with strong longitudinal ridges, apparently corresponding to alternate interstriae; prosternal keel emarginate at base; prosternal lobe short, apically truncate; mesoventrite narrowly produced at middle; propygidium slightly shorter than pygidium along midline, with gland openings near anterolateral corners; pygidium equilaterally subtriangular, apex rounded, simple; each trochanter with single long seta; femora narrow; protibia rounded apically, lacking marginal teeth, with marginal spines inserted only along apical half of edge; protibial spurs present, reduced; meso- and metatibiae thin, simple, with single longitudinal stria along inner edge, completely lacking teeth or spines along outer margin; all tarsi slightly compressed, with slightly spatulate ventral setae; male not known; female T8 divided; S8 forming a single plate, basal baculi articulated with basolateral corners, convergent, separate at base; S9 not evident, though elongate articulating strap from median apex of S8 is present; T10 not observed; valvifers paddle-shaped, basal paddles just over one-third entire valvifer length; coxites over two-thirds valvifer length, apically tridentate, with lateral teeth rather weak; gonostyle slightly shorter than median tooth, setose; bursa copulatrix membraneous, lacking sclerites, not obviously expanded; spermatheca forming a gradually enlarged, elongate sac, an elongate, slightly kinked spermathecal gland inserted near its midpoint.

#### Remarks.

**Figure 7.**
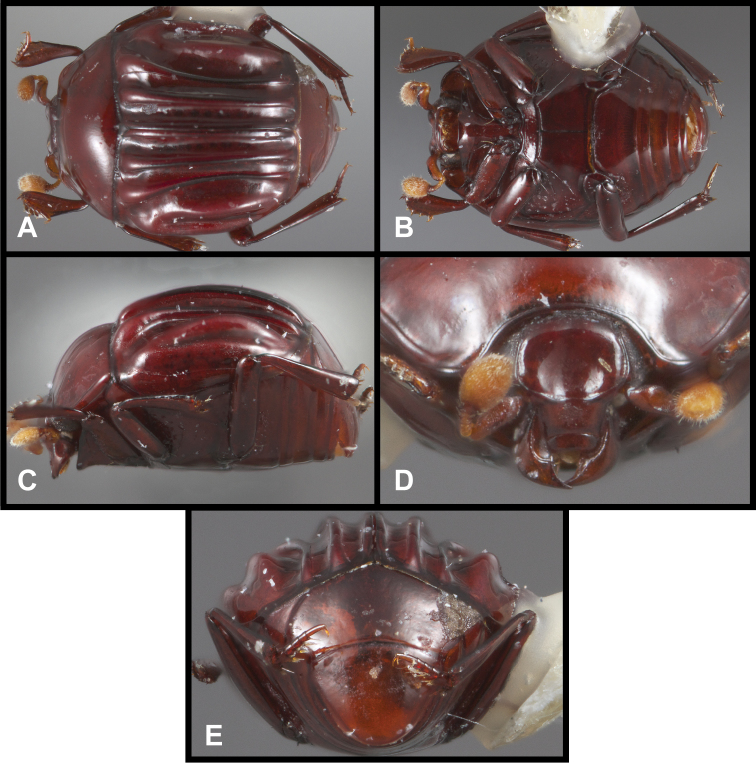
*Pluricosta onthophiloides*. **A** Dorsal habitus **B** Ventral habitus **C** Lateral habitus **D** Anterior view of head **E** Posterior view of pygidia.

This genus is unmistakable in its elevated, longitudinal elytral ridges (Fig. 7). In our recent analysis of Exosternini relationships (Caterino and Tishechkin in review), it is placed as sister to a group containing *Mecistostethus* and *Lacrimorpha*, described below.

#### Etymology.

The name of this genus refers to the series of ridges on the elytra; feminine.

### Pluricosta onthophiloides sp. n.

http://zoobank.org/501486D6-3BB6-45A5-91D6-D63EE18914C7

http://species-id.net/wiki/Pluricosta_onthophiloides

Fig. 7 , Map 1

#### Type locality.

PANAMA: Darién, Cana Biological Station [7.755°N, 77.685°W].

#### Type material.

**Holotype female:** “PANAMA: Darién, Cana Biological Station, Serranía de Pirre, 1250 m, 7°45'18"N, 77°41'6"W, 07-09 Jun 1996; J.Ashe, R.Brooks, PAN1AB96 110 ex: flight intercept trap” / “SM0034338 KUNHM-ENT” (SEMC).

#### Description.

***Size range*:** Length 1.7 mm; width 1.5 mm; ***Body*:** body round, strongly convex, with strong elytral ridges, rufescent, glabrous. ***Head*:** frons slightly longer than wide, depressed along midline, smooth, with complete frontal stria more or less rounded across front; epistoma depressed along midline, weakly emarginate apically; labrum about 4× as wide as median length, shallowly emarginate apically; mandibles with weakly arcuate incisor edges lacking basal teeth; antennal scape slightly wider near base, narrowed apically, with longitudinal carina along inner anterior edge; funicle shorter than scape, weakly widening apically, antennomere 8 short, cupuliform, not disc-like; antennal club slightly elongate oval, largely tomentose, with only indistinct subapical sensory patches. ***Pronotum*:** pronotum rather strongly convex, sides narrowed evenly from base to apex, only faintly sinuate at base and middle; marginal pronotal stria complete along lateral and apical margins; sublateral stria present along entire lateral margin, just curving mediad anteriorly, pronotal disk shallowly depressed along its inner edge; pronotal disk with two gland openings on each side, anterior opening simple, along anterior margin behind eye, posterior opening with secondary annulus, situated directly posterad anterior opening, just in front of midline; prescutellar impression absent; posterior margin of disk simple. ***Elytra*:** elytra dominated by several strong longitudinal ridges, epipleuron with single submarginal stria, continued along apical margin to apex of 2^nd^ dorsal stria; outer subhumeral stria complete; other dorsal striae very fine, running near upper edge of elevated ridges, clearly visible only near apices; apices of 3^rd^-4^th^, and 5^th^-sutural striae joined along posterior margin. ***Prosternum*:** prosternal keel shallowly but subacutely emarginate at base, carinal striae obsolete basally, joined by anterior arch short of presternal suture; prosternal lobe less than half as long as keel, apically truncate, lacking marginal stria. ***Mesoventrite*:** mesoventrite narrowly produced at middle, with complete marginal stria, disk shallowly depressed behind. ***Metaventrite*:** mesometaventral stria well impressed, coincident with mesometaventral suture; postcoxal stria directed laterad toward middle of metepisternum, ending freely; lateral metaventral stria running obliquely toward outer third of metacoxa, slightly abbreviated apically. ***Abdomen*:** 1^st^ abdominal ventrite with complete anterior marginal stria continued by postmetacoxal stria which curves laterad behind coxa, ending freely; ventrites 2-4 impunctate; propygidium only slightly shorter than pygidium along midline, smooth, with inconspicuous gland openings near anterolateral corners; pygidium similar in basal width and midline length, apex rounded, simple. ***Legs*:** each trochanter with single long seta; femora rather narrow, metafemur particularly elongate; protibia gradually widened to rounded apical half, lacking marginal teeth, with marginal spines inserted only along apical half of edge; protibial spurs present, slightly reduced; meso- and metatibiae thin, simple, with single longitudinal stria along inner edge, completely lacking teeth or spines along outer margin; all tarsi slightly compressed, with slightly spatulate ventral setae. ***Male*:** not known.

#### Remarks.

This species is known only from the female type specimen. Capture of a male would be very helpful to assessing its relationships.

#### Etymology.

The name of this species refers to its superficial resemblance to the histerid genus *Onthophilus* Leach, owing to the parallel ridges on the elytra.

### Pyxister gen. n.

http://zoobank.org/EFD5583E-6027-4D18-A2D6-177C65F1DA89

http://species-id.net/wiki/Pyxister

#### Type species.

*Pyxister devorator* sp. n.

#### Description.

***Size range*:** Length 2.4–2.8 mm; width 1.6–1.9 mm; ***Body*:** body elongate, subcylindrical, sides parallel, rufobrunneus, variably punctate, glabrous. ***Head*:** frons weakly to strongly depressed at middle, subangulate at sides in front of eyes, frontal stria complete to strongly reduced, supraorbital stria present, detached; epistoma and labrum varied; mandibles strongly toothed; mouthparts rather strongly recessed in oral cavity; submentum flat, produced in front; mentum subquadrate, nearly as long as broad, bearing sparse long setae; ultimate labial palpomeres elongate, slightly compressed; cardo glabrous, stipes with few long setae on lateral margin; ultimate maxillary palpomere slightly compressed; antennal scape elongate, curved, widest near midpoint; funicle shorter than scape, widening from antennomere 4-8, 8^th^ antennomere cupuliform, more or less disclike; antennal club short, tomentose, with single, slightly elongate, axial sensory patch on ventral surface and longer patch on dorsal surface. ***Pronotum*:** pronotal sides more or less straight, slightly convergent to apex; prescutellar impression faintly impressed to obsolete; pronotal disk with two median gland openings on each side, one very close to margin behind eye, one posterad just behind pronotal midpoint; marginal pronotal stria present on lateral and anterior margins, may be complete or interrupted at sides; submarginal pronotal stria may be present at sides. ***Elytra*:** elytra slightly depressed along suture; epipleuron with single, complete marginal stria, outer subhumeral stria interrupted at middle, inner subhumeral stria absent, striae 1-5 present, 1 and 5 may be abbreviated, sutural stria complete. ***Prosternum*:** prosternal keel emarginate at base, carinal striae present, more or less complete, convergent anterad, joined in anterior arch; prosternal lobe short, slightly deflexed, marginal stria present. ***Mesoventrite*:** mesoventrite wide, short, with marginal stria fine, close to edge, may be interrupted; mesometaventral stria strongly angulate forward nearly to margin. ***Metaventrite*:** postmesocoxal stria poorly developed, short; lateral metaventral stria extending from inner corner of mesocoxa toward middle of metacoxa, abbreviated or not. ***Abdomen*:** 1^st^ abdominal ventrite with single, oblique lateral stria; propygidium slightly convex, about two-thirds length of pygidium, gland openings may be visible near anterolateral corners; pygidium moderately to strongly convex, apical margin rounded. ***Legs*:** trochanters with single long seta; femora moderately elongate; protibia with outer margin rounded, moderately strongly dentate, with 5-6 spinose teeth; two protibial spurs present, meso- and metatibiae narrow to moderately widened to apex, mesotibia with entire margin spinose, metatibia spinose toward apex; protarsi (of male only?) with spatulate ventral setae. ***Male genitalia*:** accessory sclerites absent; T8 with narrow subacute apical emargination, ventral apodemes well developed but separated beneath, basal membrane attachment line intersecting slightly sinuate basal emargination; S8 with halves approximate in basal half, apical guides increasingly wide toward apex, rather abruptly narrowed to narrowly rounded apices, with only very fine inconspicuous setae; T9 with basal apodemes short, apex narrowly subacute, ventrolateral apodeme weakly hooked; T10 undivided; S9 desclerotized along midline, with deep apical emargination, apical flange interrupted, apical corners produced; tegmen with sides subparallel in basal two-thirds, slightly widened to subquadrate, subtruncate apex, lacking medioventral process; median lobe simple, about one-third tegmen length; basal piece short, with strong apicoventral articulating process. ***Female genitalia*:** T8 entire, with narrow apical emargination; S8 undivided, only emarginate apicolaterally, basal baculi strongly, arcuately convergent at base; S9 present, short, connected to S8 by sclerotized strap; T10 entire; valvifers gradually expanded to base, paddles about half total length; coxites large, about two-thirds valvifer length, strongly bidentate; gonostyle slightly shorter than median tooth, setose; bursa copulatrix entirely membraneous, weakly expanded; spermatheca globose; spermathecal gland not evident in preparation.

#### Remarks.

**Figure 8.**
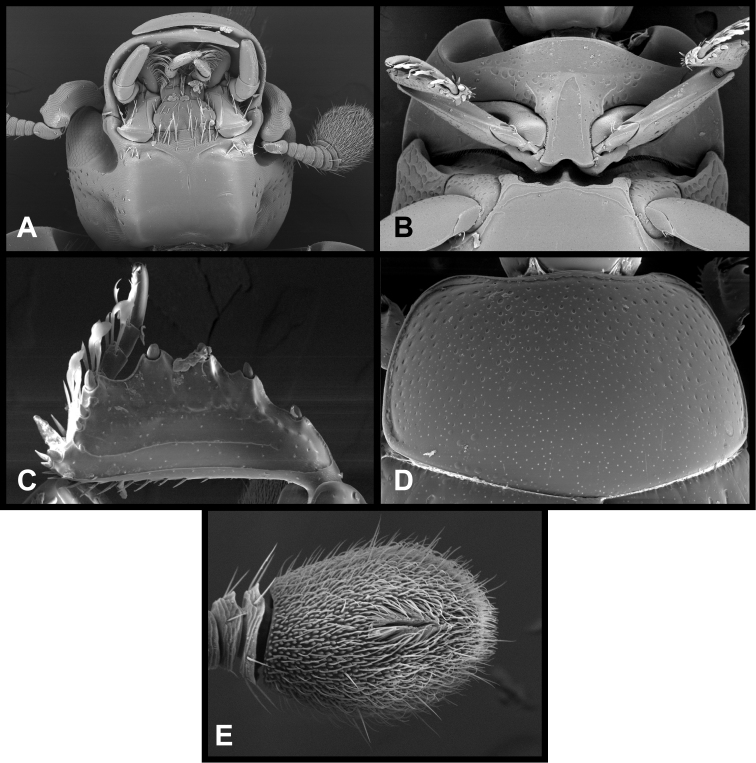
*Pyxister devorator*, SEMs showing generic characters. **A** Head, ventral view **B** Pro- and mesosterna **C** Protibia and protarsus, posterior view **D** Pronotum **E** Antennal club, dorsal view.

**Figure 9.**
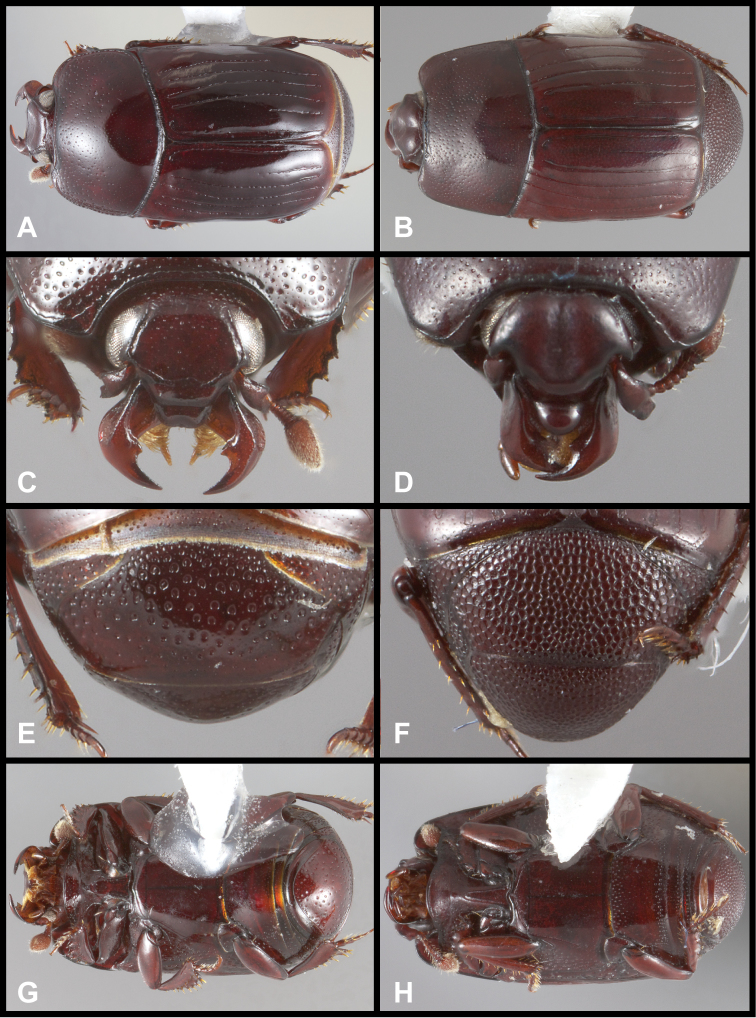
*Pyxister* spp. **A** *Pyxister devorator*, dorsal habitus **B** *Pyxister labralis*, dorsal habitus **C** *Pyxister devorator*, head, anterior view **D** *Pyxister labralis*, head, anterior view **E** *Pyxister devorator*, pygidia, posterior view **F** *Pyxister labralis*, pygidia, posterior view **G** *Pyxister devorator*, ventral habitus **H** *Pyxister labralis*, ventral habitus.

**Figure 10.**
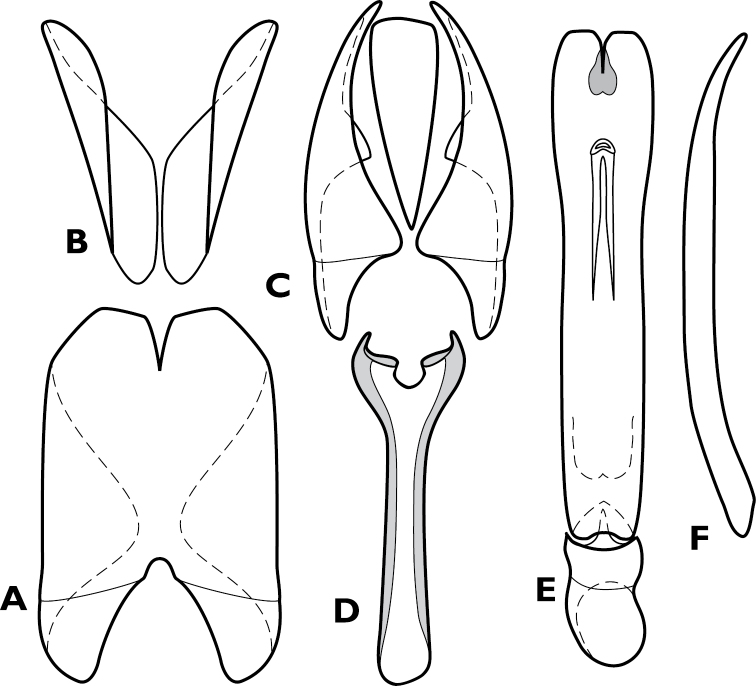
*Pyxister devorator*, male genitalia. **A** 8^th^ tergite **B** 8^th^ sternite **C** 9^th^ and 10^th^ tergites **D** 9^th^ sternite **E** Aedeagus, dorsal view **F** Aedeagus, lateral view.

The cylindrical body shape of *Pyxister* (Fig. 9A–B) will help separate it from most other Neotropical Exosternini. However, there are scattered species in many larger genera, as well as a few genera (*Yarmister* Wenzel and *Megalocraerus* Lewis, in addition to the new genus *Chapischema* described below), in which a similar body form can be found. The genus can be separated from these by the combination of subangulate frons (Fig. 9C–D), dentate mandibles, antennal club lacking annuli but with small longitudinal sensory patch (Fig. 8E), second median pronotal gland openings strongly displaced posterad, sutural elytral interval impressed, prosternal keel distinctly emarginate (Fig. 9G–H), and the mesometaventral stria nearly reaching anterior mesoventral margin, disrupting or interrupting marginal stria. It is unfortunate that the male is known for only one of the species, but unusual genitalic characters (Fig. 10) include the lack of accessory sclerites, apically broadened tegmen, lack of medioventral tegmenal process, and strong apicomedial process of the basal piece. In the female (only *Pyxister labralis*), the undivided 8^th^ sternite is unusual among Neotropical Exosternini. In our recent analysis of Exosternini relationships (Caterino and Tishechkin in review), *Pyxister* emerges from within the poorly-defined ‘scutellar impression group’, close to species with which it shares few obvious characters. Its relationships remain to be fully resolved.

#### Etymology.

Pyxis = canister or casket, referring loosely to the cylindrical body form, masculine.

#### Key to species of *Pyxister*

**Table:** 

1	Lateral submarginal pronotal stria present, complete along side; labrum flat, emarginate (Fig. 9C)	*Pyxister devorator*
–	Lateral submarginal pronotal stria absent; labrum strongly swollen (Fig. 9D)	*Pyxister labralis*

### Pyxister devorator sp. n.

http://zoobank.org/2A6B1DE9-A768-4F79-B0B2-038457E20A67

http://species-id.net/wiki/Pyxister_devorator

**Map 2.**
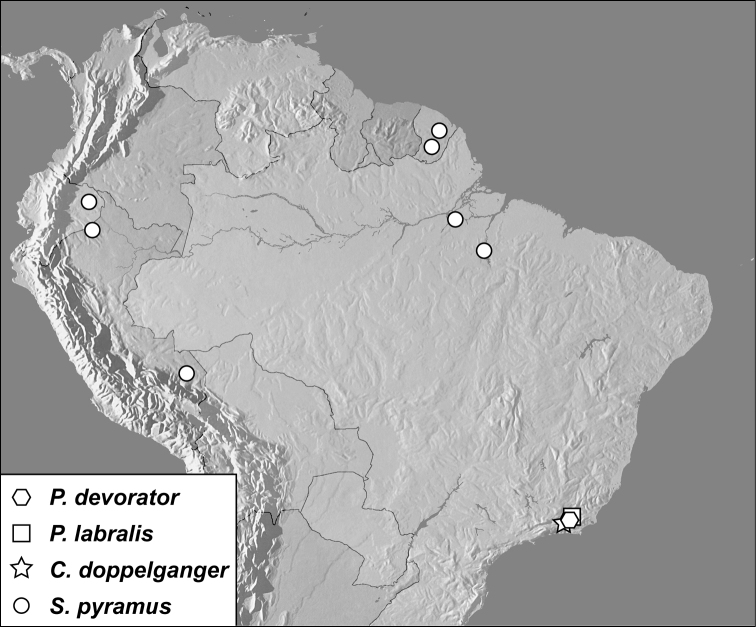
Specimen records of *Pyxister* spp., *Chapischema doppelganger*, and *Scaptorus pyramus*.

Figs 8 , 9A , 10 , Map 2

#### Type locality.

BRAZIL: Rio de Janeiro, 17km E Nova Friburgo [22.3844°S, 42.5583°W]

#### Type material.

**Holotype male:** “**BRASIL:** **RIO DE JANEIRO**, 17km E Nova Friburgo, 22°23'04"S, 42°33'30"W, 750m, 29.I.2000, F.Génier & S. Ide, secondary mountain Atlantic for. ex. f.i.t., day 4-9, **FG2000-58**” / “Caterino/Tishechkin Exosternini Voucher EXO-00159” (CMNC). **Paratypes** (4): 1: same data as type; 3: same locality as type, but 21.i.2000, FG2000-09 (CMNC, MSCC).

#### Diagnostic description.

Length: 2.5–2.8 mm, width: 1.6–1.8 mm; as for generic description, with the following specific characters: frontal disk depressed at middle, with fine ground punctation especially at sides, with very few larger secondary punctures intermingled; frontal stria complete, slightly sinuate anteriorly; frontoclypeal suture indicated by fine, complete impressed line; epistoma broad, more or less flat, apical margin straight; labrum about twice as wide as long, slightly narrowed to weakly rounded apex, basally flat, but increasingly broadly depressed toward apex; pronotal sides weakly outwardly rounded, anterior emargination very faintly produced at middle, with marginal stria complete along lateral and anterior margins, submarginal stria complete along side, curving inward at front, coarsely crenulate throughout; pronotal disk with secondary punctures almost throughout, absent only mediobasally, separated by approximately their diameters; elytral epipleuron with single, complete epipleural stria rather distant from lateral margin, outer subhumeral stria more or less complete but interrupted near middle and slightly abbreviated at base, inner subhumeral stria absent, 1^st^ dorsal stria obsolete in apical third, 2^nd^–4^th^ striae complete, 5^th^ stria obsolete in basal half, sutural stria complete, rather deeply impressed along suture; marginal mesoventral stria complete or nearly so, though weak medially and crowded by mesometaventral stria; meso- and metatibiae widened to apex, apical width about 3× basal width, both strongly spinose; propygidium with fine ground punctation interspersed with secondary punctures irregularly separated by 1–1.5× their diameters; pygidium with secondary punctures generally smaller and sparser, tending to be concentrated and more deeply impressed along lateral margins.

#### Remarks.

The two species of *Pyxister* appear clearly related, but at the same time show some remarkable differences, particularly in the structure of the head. The convex frons and strongly swollen labrum of *Pyxister labralis* (Fig. 9D) are not at all indicated in *Pyxister devorator*, which has a more or less flat frons, and a labrum which is slightly depressed and emarginate apically (Fig. 9C). Other distinguishing characters of *Pyxister devorator* are its complete lateral submarginal pronotal stria, apically abbreviated 1^st^ dorsal elytral stria, and its sparsely punctate pygidia (Fig. 9E).

#### Etymology.

The name of this species means ‘devourer’, alluding to its strong mandibles.

### Pyxister labralis sp. n.

http://zoobank.org/119C0416-4BD1-44CC-86E0-2F9507259043

http://species-id.net/wiki/Pyxister_labralis

Figs 9B–D , Map 2

#### Type locality.

BRAZIL: Rio de Janeiro, Sans Souci [22.2833°S, 42.5206°W].

#### Type material.

**Holotype female:** “Brasil, Rio de Janeiro, Nova Friburgo, Sans Souci, 9-15/XI/2009, E. Grossi (*leg.*)” / “Interceptação de vôo (FIT)” / “Caterino/Tishechkin Exosternini Voucher EXO-00749” (UFPR).

#### Diagnostic description.

Length: 2.4 mm, width: 1.7 mm; as for generic description, with the following specific characters: frons strongly transversely produced along anterior margin, depressed along midline basad anterior ridge, with antennal insertions deeply incised, frontal stria present only along inner margins of eyes, frontal disk impunctate; frontoclypeal suture not evident; epistoma recessed below frontal margin; labrum very strongly swollen, produced anterad; pronotal sides straight, parallel; marginal pronotal stria interrupted behind eyes, submarginal pronotal stria absent; pronotum very weakly tuberculate at posteromedian gland openings, disk with small secondary punctures almost throughout, absent only mediobasally, separated by approximately their diameters; elytral epipleuron with single, complete epipleural stria rather distant from lateral margin, outer subhumeral stria more or less complete but interrupted near middle and slightly abbreviated at base, inner subhumeral stria absent, 1^st^-4^th^ dorsal striae complete, 5^th^ stria obsolete in basal half, sutural stria complete, sutural intervals weakly depressed; mesoventrite with marginal stria distinctly interrupted; meso- and metatibiae rather slender, no more than twice as wide apically than basally, finely spinose; propygidium and pygidium completely coarsely reticulopunctate.

#### Remarks.

This species is easy to distinguish from its only congener by the unique shape of the frons and especially the labrum (Fig. 9D), both being strongly produced. In addition, *Pyxister labralis* lacks a submarginal pronotal stria, has the 1^st^ dorsal elytral stria entire, and has the propygidium and pygidium densely reticulopunctate (Fig. 9F).

#### Etymology.

This species’ name refers to its very distinctive, strongly convex labrum.

### Chapischema gen. n.

http://zoobank.org/536DA99C-5A83-4696-95CD-7F128E8746C5

http://species-id.net/wiki/Chapischema

#### Type species.

*Chapischema doppelganger* sp. n.

#### Description.

This genus differs from other Exosternini in the following combination of characters: body elongate, cylindrical, parallel-sided, glabrous; frons subangulate in front of eyes, moderately produced above antennal insertions, weakly depressed at middle; epistoma convex, apex truncate; labrum about twice as wide as long; left mandible with outwardly arcuate incisor edge, right mandible with small acute basal tooth; submentum slightly depressed, outwardly arcuate along anterior margin; mentum subtrapezoidal, very narrowly emarginate apically, sparsely setose; cardo glabrous, smooth; stipes with few long setae; all palpi relatively stout, ultimate palpomeres, particularly maxillary palps, thickened and with numerous conspicuous punctures; antennal scape elongate, sides sinuate, narrowed at middle in anterior aspect; funicle gradually but slightly widened to apex, 8^th^ antennomere as long as preceding antennomeres; antennal club small, about as long as preceding 4 antennomeres, lacking complete annuli, sensoria poorly defined, apparently with two widely interrupted annuli close to dorsal apex; pronotal disk with only single distinct pair of gland openings, present between anterior margin and anterior submarginal stria; prescutellar impression absent; prosternal keel very shallowly emarginate at base; prosternal lobe slightly deflexed; anterior margin of mesoventrite broadly emarginate, but very weakly produced at center; propygidium rather long, more or less flat, with small gland openings near anterolateral corners; pygidium slightly longer than propygidium along midline, apical margin simple, rounded; protrochanter glabrous, meso- and metatrochanters each with two very short apical setae; femora simple; protibia with outer margin rounded, weakly dentate, strongly spinose; protibial spurs very short; protarsus with ventral setae simple; meso- and metatibiae elongate, outer margins with spinose, mesotibia faintly dentate; meso- and metatarsi short, each with single pair apicoventral spines; male genitalia with accessory sclerites present; T8 rather short; S8 halves approximate only at base, inner margins divergent to apex, apically with very fine, inconspicuous setae; T9 with prominent, strongly hooked ventrolateral apodemes, dorsal lobes more strongly sclerotized along sides; T10 divided; S9 with apical emargination distinct, apical flange interrupted; tegmen with very large medioventral process; median lobe simple; female not known.

#### Remarks.

**Figure 11.**
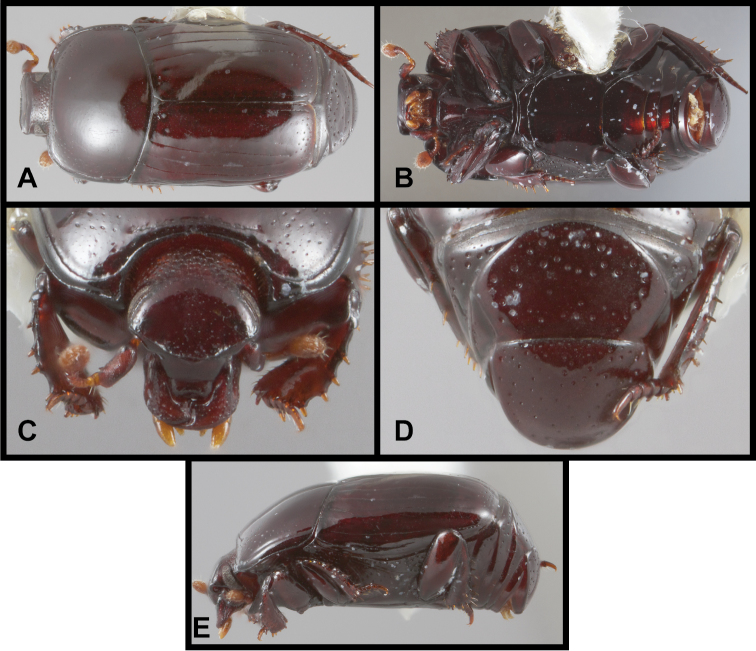
*Chapischema doppelganger*. **A** Dorsal habitus **B** Ventral habitus **C** Head, anterior view **D** Pygidia, posterior view **E** Lateral habitus.

**Figure 12.**
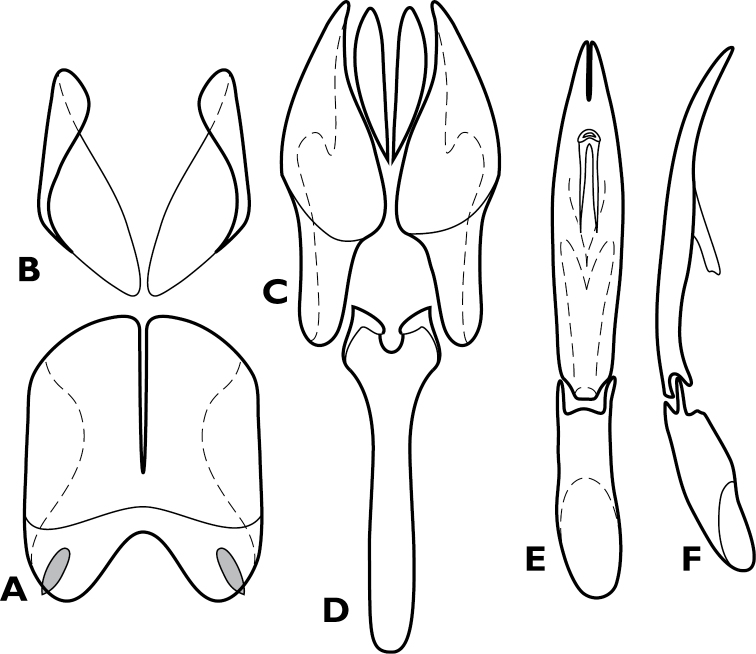
*Chapischema doppelganger*, male genitalia. **A** 8^th^ tergite **B** 8^th^ sternite **C** 9^th^ and 10^th^ tergites **D** 9^th^ sternite **E** Aedeagus, dorsal view **F** Aedeagus, lateral view.

In body form *Chapischema* is generally very similar to the preceding new genus, *Pyxister* (and likewise to *Megalocraerus*, and various other rare cylindrical Neotropical Exosternini). Among such taxa, it can best be recognized by its carinate frontal stria (Fig. 11C), abbreviated epipleural stria, and mesoventral margin, which is simultaneously broadly emarginate and narrowly produced at the middle (Fig. 11B). Generic status is supported principally by its unique male genitalia, with a very prominent medioventral tooth on the tegmen (Fig. 12F). It is resolved as closely related to *Scaptorus* and *Enkyosoma* in our forthcoming analysis of Exosternini relationships (Caterino and Tishechkin in review). However, the three are very different in appearance and this result cannot be regarded with great confidence. *Chapischema* shows no phylogenetic affinity with the superficially very similar *Pyxister*.

#### Etymology.

The name of this genus is from the Greek, meaning ‘pill-shaped’; feminine.

### Chapischema doppelganger sp. n.

http://zoobank.org/F9C0BCD3-D1F0-4E95-A822-7A1ADDDC4C92

http://species-id.net/wiki/Chapischema_doppelganger

Figs 11 –12 , Map 2

#### Type locality.

BRAZIL: Rio de Janeiro, 17km E Nova Friburgo [22.3844°S, 42.5583°W].

#### Type material.

**Holotype male:** “**BRASIL:** **RIO DE JANEIRO**, 17km E Nova Friburgo, 22°23'04"S, 42°33'30"W, 750m, 29.I.2000, F.Génier & S. Ide, secondary mountain Atlantic for. ex. f.i.t., day 4-9, **FG2000-58**” / “Caterino/Tishechkin Exosternini Voucher EXO-00013” (CMNC).

#### Description.

***Size range*:** Length 2.2 mm; width 1.3 mm; ***Body*:** body elongate, cylindrical, parallel-sided, rufobrunneus, ground punctation rather inconspicuous, glabrous. ***Head*:** frons rather broad, subangulate at sides in front of eyes, moderately produced in front above antennal insertions, beneath complete, subangulate frontal stria, weakly depressed at middle, with sparse ground punctures and very faint microsculpture within frontal depression; supraorbital stria weak but complete, narrowly detached from frontal stria at sides; epistoma convex, apex truncate; labrum about twice as wide as long, apex arcuate, but dorsal surface increasingly depressed to apex, making it appear somewhat emarginate; mandibles coarsely punctate on sides. ***Pronotum*:** pronotal sides subparallel in basal half, slightly arcuately narrowing to apex; marginal pronotal stria present on lateral and anterior margins, but narrowly interrupted behind eye, anterior portion continuous with lateral submarginal stria, which is complete and deeply impressed along sides; detached anterior submarginal stria transversely impressed behind head; pronotal disk with only single distinct pair of gland openings, located between anterior margin and free ends of transverse anterior submarginal stria; pronotal disk rather strongly convex, with few, small sparse secondary punctures near anterolateral corners; prescutellar impression absent. ***Elytra*:** elytral epipleuron with single epipleural stria present in basal half only, obsolete apically, outer subhumeral stria complete, inner subhumeral stria absent, dorsal striae 1-3 complete, rather crowded toward side, 4^th^ and 5^th^ striae absent, sutural stria thin, fragmented, obsolete in basal half; elytral disk with sparse secondary punctures in basal eighth. ***Prosternum*:** prosternal keel very shallowly emarginate at base, carinal striae complete, close together at middle, divergent anteriorly and posteriorly, free at base, united in narrow arch in front; short secondary striae present between carinal striae and procoxae; lateral prosternal striae weakly divergent; prosternal lobe very slightly deflexed, about two-thirds as long as keel, marginal stria present medially, divergent from edge, abbreviated at sides. ***Mesoventrite*:** anterior margin of mesoventrite broadly emarginate, but very weakly produced at center, marginal stria complete; mesometaventral stria coarsely crenulate, arched slightly forward onto basal third of mesoventrite. ***Metaventrite*:** postmesocoxal stria curved loosely behind coxa, extending nearly to mesepimeral-metepisternal corner; lateral metaventral stria curved laterad posteriorly toward posterior sixth of metepisternum; metaventral disk impunctate at middle, with few coarse secondary punctures at sides. ***Abdomen*:** 1^st^ abdominal ventrite with lateral striae obliquely impressed along inner edge of metacoxa; ventrites 2-4 with few coarse punctures only at extreme sides; propygidium rather long, about two-thirds as long as broad, more or less flat, with small gland openings near anterolateral corners, disk with few small, very irregularly sparse secondary punctures; pygidium elongate, about one third longer than basal width, slightly longer than propygidium along midline, apical margin rounded, disk weakly depressed along sides, punctation similar to but finer and sparser than that of propygidium. ***Legs*:** protrochanter glabrous, meso- and metatrochanters each with two very short apical setae; femora simple; protibia with outer margin rounded, weakly 5-dentate, each tooth with moderately strong spine; posterior surface rather coarsely dimpled; protibial spurs very short; protarsus with ventral setae simple; meso- and metatibiae elongate, widened apically to about twice basal width, outer margins with 4-5 rather strong spines, mesotibia in particular faintly dentate; meso- and metatarsi short, each with single pair apicoventral spines. ***Male genitalia*** (Fig. 12): accessory sclerites present; T8 rather short, with sides subparallel, apex very deeply, narrowly emarginate, basal membrane attachment line complete distad basal emargination, ventrolateral apodemes moderately developed, well separated beneath; S8 halves approximate only at base, inner margins divergent to apex, apical guides widest near apex, with very fine inconspicuous setae; T9 with broad basal apodemes, prominent, strongly hooked ventrolateral apodemes, dorsal lobes more strongly sclerotized along sides; T10 weakly sclerotized, completely divided; S9 with sides sinuate, subparallel, apical emargination distinct, rounded, apical flange interrupted, apical corners moderately prominent; tegmen widest near middle, slightly narrowed to base, more strongly narrowed to apex, with very large medioventral process; median lobe simple, about half tegmen length; basal piece long, about two-thirds tegmen length.

#### Remarks.

The superficial similarity of this species with *Pyxister devorator*, also exclusively known from the same collecting event near Nova Friburgo, Brazil, is remarkable. Despite the apparent similarity, they do differ in several external characters, and the male genitalia differ in numerous substantial characters. Their external similarity must be considered an extreme convergence.

#### Etymology.

This species’ name refers to its extreme similarity to the sympatric *Pyxister devorator*.

### Scaptorus gen. n.

http://zoobank.org/4B2498BC-56C0-4E99-9AFC-51866CB77347

http://species-id.net/wiki/Scaptorus

#### Type species.

*Scaptorus pyramus* sp. n.

#### Description.

This genus differs from other Exosternini in the following combination of characters: body elongate, sides subparallel, distinctly convex; frons widened to rounded anterior corners produced over antennal bases; epistoma bituberculate along anterior margin; labrum with apical margin truncate, weakly produced and carinate; left mandible with straight, edentate incisor edge, right mandible curved to apex, with small, acute basal tooth; submentum rather short, slightly depressed, weakly produced anteriorly into oral cavity; mentum broadly subquadrate, weakly emarginate apically, labial palpi 3-segmented, basal palpomere very short, penultimate and ultimate palpomeres rather thin and elongate; maxillary cardines very smooth, stipes faintly microsculptured, with few scattered setae, maxillary palpi 4-segmented, basal palpomere short, 2^nd^ and 3^rd^ palpomeres subequal, ultimate palpomere about twice as long as penultimate, simply fusiform; antennal scape elongate, very slightly widened toward apex, with few setae on anterior surface; funicle slightly shorter than scape, widened slightly to cupuliform 8^th^ antennomere; antennal club elongate oval, about as long as funicle, tomentose, with median and subapical annuli crowded into apical half, both interrupted on dorsal surface, free ends enlarged into sensory patches, the apical-most more so; pronotal gland openings not evident; pronotal sides subparallel, abruptly bent to apical corners; transverse elevated carina present behind and parallel to anterior pronotal margin, curving posterad briefly at sides, abruptly bent to lateral margin behind anterior corner; prosternal keel overlapped at base by projecting mesoventrite, weakly convex, short, anteriorly displaced by prominent prosternal lobe, which is strongly produced, deflexed, with raised median ridge; mesoventrite subtrapezoidal, projecting anteriorly; mesometaventral stria absent; propygidium transverse, with small gland openings in anterolateral corners; pygidium about one third wider than long, apical margin simply rounded; protrochanter lacking setae, meso- and metatrochanters with pair of very short setae near apex of posterior edge; profemur rather narrow, anterior edge sinuate; protibia narrow at base, outer margin more or less rounded apically, weakly dentate, strongly spinose; protibial spurs present, short; protarsus somewhat laterally compressed, bearing spatulate ventral setae in both sexes; meso- and metafemora narrow; meso- and metatibiae slightly widened to apices, bearing few thin spines toward apex of outer margins; male genitalia with paired accessory sclerites present; T8 with ventral apodemes nearly meeting along midline; S8 with halves divergent apically, apical guides well developed, with subapical setae; T9 with broad, blunt ventrolateral apodemes; T10 completely divided; S9 head broad, with prominent lateral flanges, apical flange low, continuous, not interrupted medially; tegmen lacking medioventral tooth or process; median lobe long, basal processes strongly differentiated; female T8 forming a single plate; S8 tripartite, basal baculi narrowly articulated with lateral plates, thin and convergent proximally; S9 well developed, about twice as long as broad, articulated with strap-shaped extension from apex of S8; T10 entire; valvifers about as long as coxites, weakly paddle-shaped, weakly expanded in basal third; coxites nearly three times as long as wide, bidentate, with median apical tooth rather thin and elongate, outer tooth weakly developed, the two well separated; gonostyle flattened, shorter than median tooth, apically setose; bursa copulatrix membranous, barely expanded; spermatheca rather short, sausage-shaped, with basal stem short, with slightly thin, very elongate spermathecal gland attached near its base.

#### Remarks.

**Figure 13.**
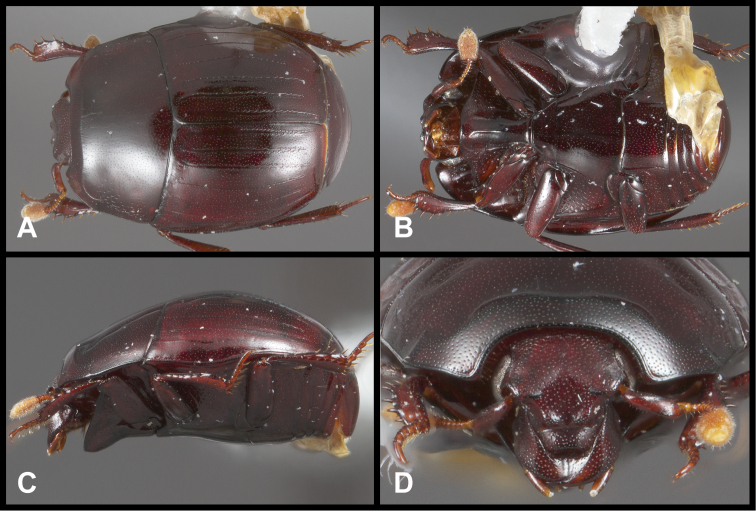
*Scaptorus pyramus*. **A** Dorsal habitus **B** Ventral habitus **C** Lateral habitus **D** Head, anterior view.

**Figure 14.**
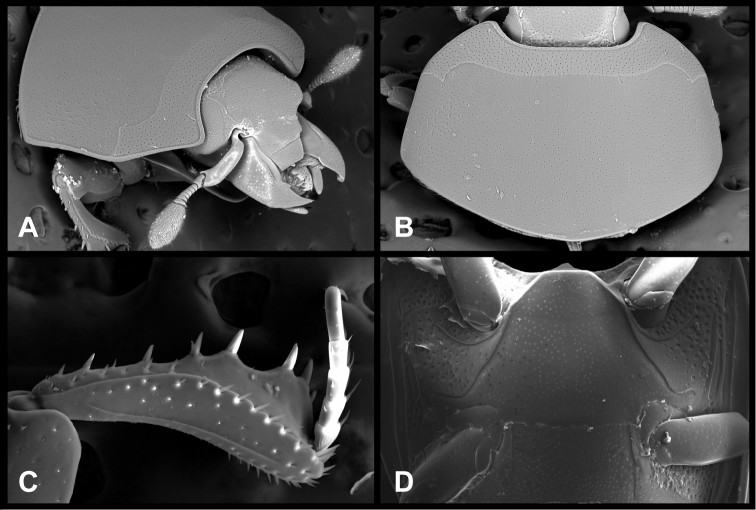
*Scaptorus pyramus*, SEMs. **A** Head and pronotum, anterolateral view **B** Pronotum **C** Protibia, anterior view **D** Meso- and metaventrites.

*Scaptorus* is characterized by numerous unique features. The transverse carina of the pronotum (Figs 13A, 14A–B) is the most obvious, immediately separating this from other Neotropical Exosternini. But in addition the bituberculate epistomal margin, projecting mesoventral margin (overlapping the reduced prosternal keel), and longitudinally ridged prosternal lobe (Fig. 13B) are also very unusual. A somewhat comparable mesosternum can be found in *Mecistostethus* Marseul, but this is almost certainly convergence; the two genera share little else. It is instead resolved as closely related to *Enkyosoma* and *Chapischema* in our recent analysis of Exosternini relationships (Caterino and Tishechkin in review).

#### Etymology.

The name of this genus translates to ‘shoulder ridge’ referring to its most diagnostic feature.

### Scaptorus pyramus sp. n.

http://zoobank.org/02EDCF2F-50F2-44DA-85CA-B9C54FD7F423

http://species-id.net/wiki/Scaptorus_pyramus

**Figure 15.**
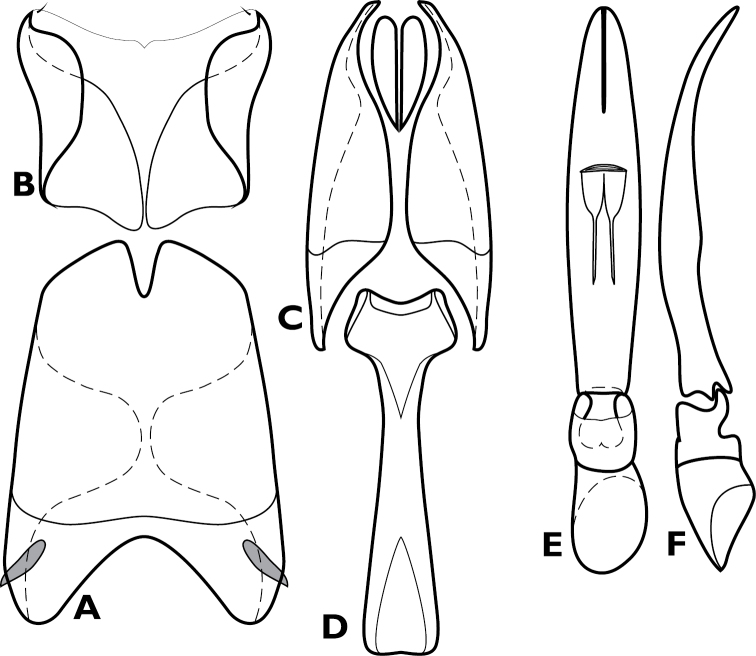
*Scaptorus pyramus*, male genitalia. **A** 8^th^ tergite **B** 8^th^ sternite **C** 9^th^ and 10^th^ tergites **D** 9^th^ sternite **E** Aedeagus, dorsal view **F** Aedeagus, lateral view.

Figs 13 – 15 , Map 2

#### Type locality.

FRENCH GUIANA: Belvèdére de Saül [3.01°N, 53.21°W].

#### Type material.

**Holotype male:** **GUYANE FRANÇAISE:** Belvèdére de Saül, point de vue. 3°1'22"N, 53°12'34"W. Piège vitre 31.xi.2010. SEAG leg.” / “Caterino/Tishechkin Exosternini Voucher EXO-01763” (MNHN). **Paratypes** (6): 5: same data as type, except 2: 24.i.2011, 2: 10.xii.2010, 1: 17.i.2011; 1: Rés. des Nouragues, Camp Inselberg, 4°05'N, 52°41'W, 8.x.2010, FIT, SEAG (CHND, FMNH, MSCC).

#### Other material.

1: **ECUADOR:** **Orellana**, Yasuní Res. Stn. on mid. Rio Tiputini. 0°40.5'S, 76°24'W, FIT, 23–30.vi.1999, A.Tishechkin (LSAM), 1: 28.vi–5.vii.1999 (LSAM). 1: **PERU:Loreto:** Campamento San Jacinto, 2°18.75'S, 75°51.77'W, 175–215m, 7.vii.1993, FIT, R. Leschen (SEMC); 1: **Madre de Dios:** CICRA Field Station, 12.55261°S, 70.11008°W, 295m, 11–13.vii.2010, blue pan trap, Chaboo Team (SEMC). 1: **BRAZIL:** **Pará:** Tucuruí, 3°45'S, 49°40'W, FIT, 27.x-9.xi.1985 (CHND); Melgaço Dist., Rio Marinau, 1°51.5'S, 51°20'W, FIT, 29.x-13.xi.1993 (CHND).

#### Description.

***Size range*:** Length 2.2-2.5 mm; width 1.9-2.1 mm; ***Body*:** body rufobrunneus, elongate, sides subparallel, distinctly convex. ***Head*:** frons nearly as long as broad, more or less flat, weakly depressed in middle, sides widened very slightly to rounded anterior corners produced over antennal bases; frontal stria fine, present close to sides and along anterolateral edges, absent from middle; supraorbital stria absent; frontal disk with fine but conspicuous ground punctation throughout, with few coarser punctures along dorsal margin, faintly microsculptured; epistoma bituberculate along anterior margin, tubercles subtended by weak carinae to ends of frontal stria, epistomal disk depressed along midline; labrum about one-third as long as broad, apical margin truncate, weakly produced and carinate above flattened supraoral surface. ***Pronotum*:** pronotal sides subparallel in basal two-thirds, abruptly bent to apical corners; pronotal gland openings not evident; marginal stria complete along lateral and anterior margins; sinuate, transverse elevated carina present one-fourth behind and parallel to anterior pronotal margin, curving posterad briefly at sides, abruptly bent to lateral margin one-third from anterior corner; ground punctation of disk fine posterad carina, markedly denser anterad carina, with few coarser, shallow punctures at sides; prescutellar impression absent. ***Elytra*:** convexity of elytra slightly greater than that of pronotum, i.e., lateral profile not a smooth curve; epipleuron smooth, with single, complete marginal stria; outer subhumeral stria carinate, forming distinct lateral margin to dorsal surface, inner subhumeral stria present only in basal half, striae 1-4 complete, 5^th^ stria obsolete in basal one-third, rarely complete, sutural stria obsolete in basal one-third; all dorsal striae rather shallowly impressed, but 4^th^, 5^th^ and sutural striae broad, at least apically, delimited on inner and outer edges. ***Prosternum*:** prosternal keel overlapped at base by projecting mesoventrite, lacking carinal striae, weakly convex, short, anteriorly displaced by prominent prosternal lobe, which is strongly produced, deflexed, with raised median ridge, marginal stria absent. ***Mesoventrite*:** mesoventrite subtrapezoidal, narrowed, projecting anteriorly, lacking marginal stria; mesometaventral stria absent. ***Metaventrite*:** postcoxal stria present, curved around mesocoxa to middle of mesepimeron; lateral metaventral stria extending from inner corner of mesocoxa toward middle of metacoxa, bent laterad apically toward metepisternum; metaventral punctation fine and sparse at middle, with few coarser punctures at sides. ***Abdomen*:** 1^st^ abdominal ventrite with ground punctation rather dense, with parallel lateral striae along inner edge of metacoxa; ventrites 2-4 simply punctate, lacking transverse striae; propygidium wide, rather short, with small gland openings in anterolateral corners; pygidium about one third wider than long, apical margin simply rounded. ***Legs*:** protrochanter lacking setae, meso- and metatrochanters with pair of very short setae near apex of posterior edge; profemur rather narrow, anterior edge sinuate, partial stria along posterior margin; protibia narrow at base, outer margin widened to more or less rounded apical half, weakly dentate, each tooth bearing thin but rather long spine; protibial spurs present, short; protarsus somewhat laterally compressed, bearing spatulate ventral setae in both sexes; meso- and metafemora narrow, slightly elongate; meso- and metatibiae slightly widened to apices, bearing few thin spines toward apex of outer margins. ***Male genitalia*** (Fig. 15): Paired accessory sclerites present; T8 with broad basal and narrower apical emarginations, line of basal membrane attachment complete, distad basal emargination, ventral apodemes well developed, nearly meeting along midline; S8 with halves nearly meeting only at base, divergent apically, apical guides well developed from base to near apex, rather abruptly narrowed to subacute apex, each side with single inconspicuous subapical seta; T9 with broad, blunt ventrolateral apodemes, apices narrow, obliquely subtruncate; T10 completely divided; S9 broad, truncate at base, narrowed toward apex, head broad, with prominent lateral flanges, apical flange low, continuous, not interrupted medially; tegmen rather narrow, widest at middle, evenly narrowed to base and apex, weakly curved in lateral aspect, lacking medioventral tooth or process; basal piece about one-third tegmen length; median lobe almost half tegmen length, gonopore rather wide, basal processes strongly differentiated, with thin proximal arms over half overall length.

#### Remarks.

The majority of specimens of this unusual species appear to bear witness to some interesting aspect of its biology. Most show distinct longitudinal scratches on the pronotal disk behind the transverse carina (Fig. 14B). What might be causing this is unclear, but we would suggest ant mandibles as a possibility. We limit the type series to those specimens from French Guiana, due to the lack of males from other localities with which to confirm identity.

#### Etymology.

The name of this species refers to pyramidal swellings on the apex of the epistoma.

### Lacrimorpha gen. n.

http://zoobank.org/1339536E-A1A2-42D6-A0CE-73B092CEE826

http://species-id.net/wiki/Lacrimorpha

#### Type species.

*Lacrimorpha glabra* sp. n.

#### Description.

***Size range*:** Length 1.8–2.2 mm; width 1.5–1.8 mm; ***Body*:** body depressed, sublimuloid, with sides rounded and pygidia variably prolonged, generally lightly colored, rufescent to rufo-brunneus, smooth, with fine ground punctation but very little secondary punctation. ***Head*:** frons and epistoma convex, prominent, frons rather broad with frontal stria complete, weakly recurved at middle; supraorbital stria fine, usually detached at sides; epistoma weakly emarginate apically; labrum short inwardly arcuate, about 4× wider than long; mandibles rather short, basal denticles on incisor very small to obsolete; submentum transversely depressed, posterolateral margins raised, apical margin produced slightly into base of oral cavity; mentum about twice as wide as midline length, sides narrowed, apex weakly emarginate; maxillary cardo glabrous, stipes with two setae along lateral margin, basal palpomere short, 2^nd^ and 3^rd^ palopmeres short, subequal, ultimate palpomere narrowed apically, about 1.5× as long as penultimate; antennal scape weakly expanded to apex, weakly carinate along inner margin, funicle narrow at base, weakly widened to 7^th^ and disc-like 8^th^ antennomere; antennal club tomentose, basal annulus obsolete, middle annulus with slightly basally expanded sensory patch on upper surface, simple on lower surface, apical annulus poorly defined, transverse. ***Pronotum*:** pronotal sides arcuate, strongly convergent to anterior corners; prescutellar impression absent; median pronotal gland openings very fine, one pair along anterior margin laterad eye, one pair behind eye about two-thirds pronotal length from anterior margin; marginal pronotal stria complete and continuous along lateral and anterior margins; submarginal stria complete laterally, not extending mediad behind head, very close to marginal stria, intervening disk narrowly convex. ***Elytra*:** elytral striation strongly reduced, single epipleural stria present, complete, diverging from margin in anterior half, outer subhumeral stria complete, nearly meeting 1^st^ dorsal stria apically, inner subhumeral stria absent, oblique humeral stria faint, 1^st^ dorsal stria more or less complete, may be slightly abbreviated basally, may be extended mediad along posterior margin of elytron, 2^nd^ and third dorsal striae weakly impressed, present in basal half or less, 4^th^ and 5^th^ striae completely absent, sutural stria usually represented only by extremely short striole at posteromedian corner of elytron, may be extended laterad by apical marginal stria. ***Prosternum*:** prosternal keel narrow, acutely emarginate at base, carinal striae weak to absent; prosternal lobe about half as long as keel, marginal stria present or absent. ***Mesoventrite*:** mesoventrite acutely produced in front, marginal stria complete, with varied fine strioles in anterolateral corners; mesometaventral stria absent. ***Metaventrite*:** mesoventrite with postmesocoxal stria present, varied in length, lateral metaventral stria absent; median portion of metaventral disk with fine ground punctation only, grading to coarser punctures laterad coxae, punctures along metaventral-metepisternal suture may coalesce into stria; metepisternum often with longitudinal stria. ***Abdomen*:** 1^st^ abdominal ventrite with single faint to abbreviated stria along inner margin of metacoxa, generally curved laterad behind coxa, disk faintly strigose at sides; ventrites 2-4 with posterior marginal stria along lateral thirds or more; ventrite 5 variously prolonged, apical margin strongly arcuate; propygidium flat or faintly depressed at sides, with basal marginal stria, complete or not; propygidial disk without obvious gland openings; pygidium with apex subacute to very prolonged and acuminate, with lateral marginal striae or not. ***Legs*:** all femora flattened and slightly expanded, arcuate on anterior and posterior edges; each trochanter with single seta; protibia with inner and especially outer edges arcuate, narrowing apically, the outer edge bearing 6-7 strong spines, denser near apex, lacking emarginations between, two apical protibial spurs present, strongly reduced, anterior surface of protibia with tarsal groove almost obsolete; protarsus of both sexes bearing spatulate ventral setae; meso- and metatibiae very narrow, parallel-sided, bearing a few thin spines toward apex of inner and outer edges; meso- and metatarsi as long or longer than corresponding tibia, with long, ventral setae that may be vaguely spatulate. ***Male genitalia*:** accessory sclerites present, basal; T8 with weakly developed ventrolateral apodemes, apical margin may be slightly desclerotized, basal membrane attachment line intersecting basal emargination; S8 divided, inner edges divergent in apical half, lateral guides weakly to moderately developed, apices narrowed, bearing a few conspicuous setae near apical corners; T9 with ventrolateral apodemes only very weakly developed, not hooked, apices narrowed, acute at inner corners; T10 weakly sclerotized, completely divided; S9 broad, sclerotized along edges, with small apical emargination and weak apical flanges; tegmen flattened, moderately broad basally, slightly narrowed apically, lacking ventromedial process; median lobe more than half as long as tegmen, with proximal apodemes prominent, abruptly narrowed at extreme proximal end; basal piece long, about half as long as tegmen, with prominent apicoventral point. ***Female genitalia*:** T8 forming a single plate, apically desclerotized, with shallow, arcuate basal emargination; S8 tripartite, with median sclerite weakly divided from lateral sclerties, basal baculi narrowly attached to lateral sclerites, evenly convergent proximally; S9 weakly sclerotized, elongate, articulated with strap-shaped extension from apex of S8; T10 broad, apically arcuate; valvifers paddle-shaped, paddles nearly one-half total length; coxites elongate, two-thirds length of valvifers, tridentate, with very prominent median tooth dwarfing teeth on either side; gonostyle long, bisetose, inserted between two lateral-most apical teeth; bursa copulatrix membranous, weakly expanded; spermatheca weakly sclerotized, approximately spherical, borne on long thin stalk inserted at base of common oviduct, with elongate, weakly spiraled spermathecal gland attached near its base.

#### Diagnosis.

**Figure 16.**
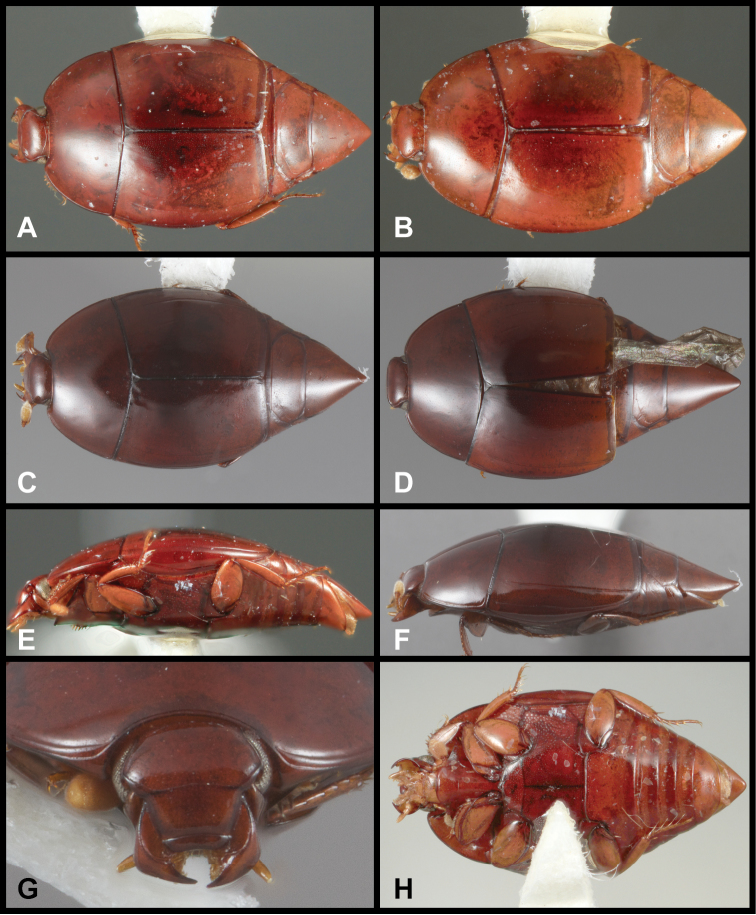
*Lacrimorpha* spp. **A** *Lacrimorpha glabra*, dorsal habitus **B** *Lacrimorpha balbina*, dorsal habitus **C** *Lacrimorpha subdepressa*, dorsal habitus **D** *Lacrimorpha acuminata*, dorsal habitus **E** *Lacrimorpha glabra*, lateral habitus **F** *Lacrimorpha subdepressa*, lateral habitus **G** *Lacrimorpha acuminata*, head, anterior view **H** *Lacrimorpha glabra*, ventral habitus.

This genus is easy to recognize based on its sublimuloid shape (Fig. 16), with the body depressed, the sides rounded, and the pygidium variably prolonged and subacute. Its convex frons is also unusual, as are the rounded, spinose protibiae, the very narrow meso- and metatibiae, the reduced elytral striation, and the almost complete lack of secondary punctation. *Lacrimorpha* is resolved as the sister group of the genus *Mecistostethus* in our recent analysis of Exosternini relationships (Caterino and Tishechkin in review).

#### Etymology.

The name of this genus means ‘tear-drop shaped’, resulting from its tapered posterior end. The genus is feminine.

#### Key to species of *Lacrimorpha*

**Table:** 

1	Postmesocoxal stria ending freely behind coxa	2
–	Postmesocoxal stria recurved anterad to mesepimeron	3
2	Lateral edge of metaventrite lacking stria near metepisternal suture; prosternal keel with weak carinal striae; marginal stria of prosternal lobe well developed at middle	*Lacrimorpha glabra*
–	Lateral edge of metaventrite with stria along anterior half of metepisternal suture; prosternal keel lacking carinal striae; marginal stria of prosternal lobe fragmented to absent	*Lacrimorpha subdepressa*
3	Basal propygidial stria complete; lateral edge of metaventrite with stria along metepisternal suture; apex of pygidium subacute, pygidium more or less equilateral, lacking lateral striae	*Lacrimorpha balbina*
–	Basal propygidial stria interrupted; lateral edge of metaventrite lacking stria near metepisternal suture; pygidium strongly prolonged, apex acuminate, with lateral strioles	*Lacrimorpha acuminata*

### Lacrimorpha glabra sp. n.

http://zoobank.org/D7C3978A-F7C9-49FF-AB69-89D1DEA948E1

http://species-id.net/wiki/Lacrimorpha_glabra

**Map 3.**
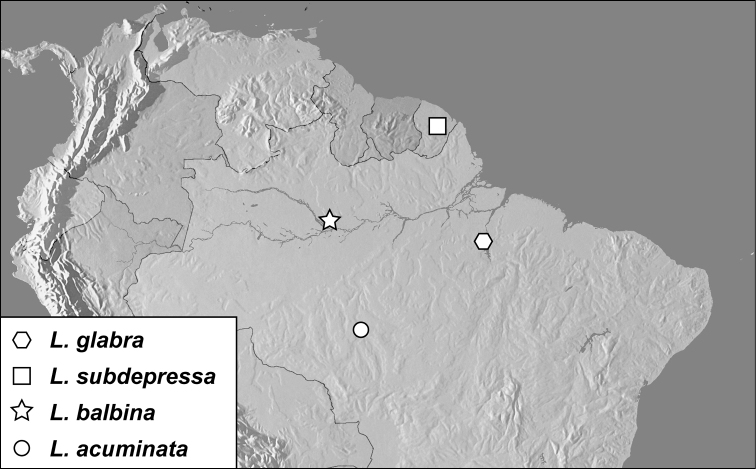
Specimen records of *Lacrimorpha* spp.

**Figure 17.**
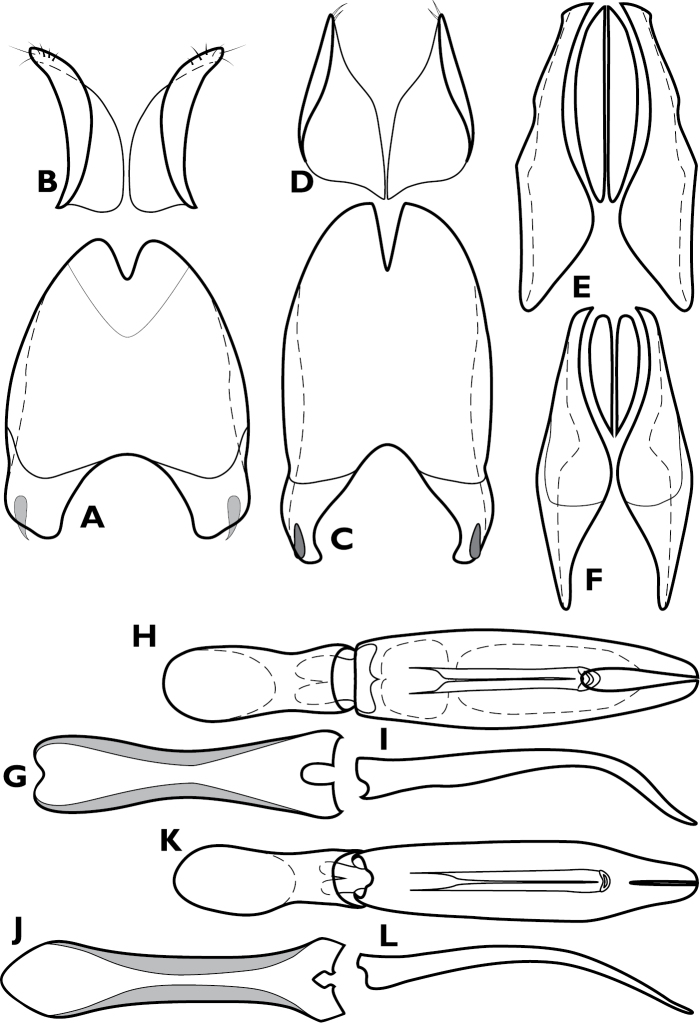
*Lacrimorpha* spp., male genitalia. **A** *Lacrimorpha glabra*, 8^th^ tergite **B** *Lacrimorpha glabra*, 8^th^ sternite **C** *Lacrimorpha acuminata*, 8^th^ tergite **D** *Lacrimorpha acuminata*, 8^th^ sternite **E** *Lacrimorpha glabra*, 9^th^ and 10^th^ tergites **F** *Lacrimorpha acuminata*, 9^th^ and 10^th^ tergites **G** *Lacrimorpha glabra*, 9^th^ sternite **H** *Lacrimorpha glabra*, aedeagus, dorsal view **I** *Lacrimorpha glabra*, aedeagus, lateral view **J** *Lacrimorpha acuminata*, 9^th^ sternite **K** *Lacrimorpha acuminata*, aedeagus, dorsal view **L** *Lacrimorpha acuminata*, aedeagus, lateral view.

Figs 16A, E, H , 17A–B, E, G–I , Map 3

#### Type locality.

BRAZIL: Pará: Tucuruí [3.75°S, 49.67°W].

#### Type material.

**Holotype male:** “Tucurui 49°40'W, 3°45'S, PARA BRESIL” / “16-29/7/1985, piége d’interception, N. Degallier” / “Caterino/Tishechkin Exosternini Voucher EXO-00189” (UFPR).

#### Diagnostic description.

Length: 2.0 mm, width: 1.8 mm; as for generic description, plus the following specific characters: body rufescent; mandibles lacking basal incisor teeth; apices of first and sutural elytral striae connected by apical marginal elytral stria; basal propygidial stria nearly complete, only narrowly interrupted at middle; fine ground punctation of pygidia relatively conspicuous; pygidium equilaterally subtriangular, apex bluntly subacute; prosternal keel with weak carinal striae in basal half only; prosternal lobe with well impressed marginal stria along middle portion; postmesocoxal stria bent laterad behind coxa, ending freely; only few punctures along metepisternal margin subserially arranged, without marginal stria on edge of mesoventrite; metepisternum itself with fragmented longitudinal stria; postmetacoxal stria not distinguishable from lateral strigosity of 1^st^ abdominal ventrite.

#### Remarks.

This species has its pygidial apex subacute (Fig. 16A), but not particularly prolonged. This in combination with the posteriorly abbreviated postmesocoxal stria and lack of lateral marginal metaventral stria will distinguish it from its congeners.

#### Etymology.

This species’ name refers to its very smooth, glabrous body surface.

### Lacrimorpha subdepressa sp. n.

http://zoobank.org/341A2D9F-B60C-44D2-BCF3-9F11984026C3

http://species-id.net/wiki/Lacrimorpha_subdepressa

Figs 16C, F , Map 3

#### Type locality.

FRENCH GUIANA: Rés. des Nouragues [4.0834°N, 52.6833°W].

#### Type material.

**Holotype female:** “Rés. Natur. des Nouragues, Camp Inselberg, 4°05'N, 52°41'W, Piège vitre, 30.ix.2010, SEAG leg.” (MNHN). **Paratypes** (4 females): 3: same locality as type, 30.xi.2010, 16.ix.2010, and 25.i.2011; 1: Rés. Natur. des Nouragues, Saut Pararé, 4°02'N, 52°41'W, FIT, 20.iv.2010, SEAG. (CHND, LSAM, MSCC).

#### Diagnostic description.

Length: 1.9–2.1 mm, width: 1.6–1.8 mm; as for generic description, plus the following specific characters: body rufobrunneus; right mandible with weak basal incisor tooth; apical marginal elytral stria absent; propygidium with basal marginal stria broadly interrupted at middle; pygidium with apex bluntly subacuminate, with sides slightly narrowed toward apex, barely longer than basal width; prosternal keel lacking carinal striae; prosternal lobe with at most weak fragments of marginal stria, may be obsolete; postmesocoxal stria bent laterad behind coxa, ending freely; metaventral disk with distinct stria along basal half to two-thirds of metepisternal suture; longitudinal metepisternal suture well developed; postmetacoxal stria not distinguishable from lateral strigosity of 1^st^ abdominal ventrite.

#### Remarks.

This species can be separated from the others in the genus *Lacrimorpha* by its slightly darker color (Fig. 16C), presence of half to two-thirds of the lateral metaventral stria, weak prosternal lobe stria, and absence of carinal striae of the prosternal keel.

#### Etymology.

The name of this species refers to its moderately subdepressed body form.

### Lacrimorpha balbina sp. n.

http://zoobank.org/F88C3135-5E9D-4966-99AE-E0B7E2E9841B

http://species-id.net/wiki/Lacrimorpha_balbina

Fig. 16B , Map 3

#### Type locality.

BRAZIL: Amazonas, Balbina [1.9553°S, 59.4580°W].

#### Type material.

**Holotype female:** “20-30/IV/88 FIT BALBINA Amazonas, Brésil” / “Caterino/Tishechkin Exosternini Voucher EXO-00190” (UFPR).

#### Diagnostic description.

Length: 1.8 mm, width: 1.6 mm; as for generic description, plus the following specific characters: body rufescent; mandibles lacking basal incisor teeth; apical marginal elytral stria absent, apical sutural striole strongly reduced; basal propygidial stria complete and close to basal margin; ground punctation of propygidium slightly more conspicuous than that of pygidium; pygidium subtriangular, slightly shorter than basal width, apex bluntly subacute; prosternal keel with weak carinal striae in basal half; prosternal lobe with fragment of marginal stria at middle; postmesocoxal stria recurved around coxa to mesepimeron; secondary punctation of sides of metaventrite very shallow and sparse; lateral stria present along metepisternal margin; metepisternum with complete longitudinal stria; 1^st^ abdominal ventrite with lateral stria present along inner edge of metacoxa and bending laterad behind coxa.

#### Remarks.

This species can be distinguished from the others in the genus by the combination of: relatively weakly produced pygidial apex (Fig. 16B), complete basal propygidial stria, and completely recurved postmesocoxal stria.

#### Etymology.

This species is named for its type locality, close to the dam of this name northeast of Manaus.

### Lacrimorpha acuminata sp. n.

http://zoobank.org/8D6F599D-6DDB-427A-805A-4775E213E61C

http://species-id.net/wiki/Lacrimorpha_acuminata

Figs 16D, G , 17C–D, J–L , Map 3

#### Type locality.

BRASIL: Mato Grosso, Fazenda São Nicolau [9.859°S, 58.215°W].

#### Type material.

**Holotype male:** “**BRASIL:** **Mato Grosso:** Mpio. Cotriguaçu, Fazenda São Nicolau, Prainha, 9°51.6'S, 58°12.9'W, flight intercept, Oct. 2009, F.Z.Vaz-de-Mello” / “Caterino/Tishechkin Exosternini Voucher EXO-01301” (CEMT).

#### Diagnostic description.

Length: 2.0 mm, width: 1.8 mm; as for generic description, plus the following specific characters: body rufescent, with ground punctation very fine and sparse; right mandible with small, acute basal incisor tooth; apical marginal elytral stria absent, apical sutural striole strongly reduced; basal propygidial stria interrupted for about one-fifth width of propygidium; ground punctation of propygidium slightly more conspicuous than that of pygidium; pygidium about one-third longer than basal width, with lateral marginal striae in basal half, apex bluntly subacute; prosternal keel lacking carinal striae; prosternal lobe with marginal stria well impressed in middle; postmesocoxal stria recurved around coxa to mesepimeron; secondary punctures of sides of metaventrite small and uniform but rather dense; lateral stria absent from metepisternal margin; metepisternum with complete longitudinal stria; 1^st^ abdominal ventrite with lateral stria difficult to distinguish from ground strigosity.

#### Remarks.

This species’ most distinctive character is its very distinctly prolonged pygidial apex (Fig. 16D). It also, like the preceding species, has its postmesocoxal stria recurved completely to the mesepisternum, but has its basal propygidial stria interrupted medially.

#### Etymology.

This species’ name refers to the acuminate pygidium.

### Crenulister gen. n.

http://zoobank.org/0B3C5374-EC9F-4A51-BA6A-FE8CF8FD7C6E

http://species-id.net/wiki/Crenulister

#### Type species.

*Crenulister grossus* sp. n.

#### Description.

***Size range*:** Length 1.7–3.2 mm; width 1.5–2.8 mm; ***Body*:** body rather broadly ovoid, variably subdepressed to rather strongly depressed, rufescent to rufopiceous. ***Head*:** frons broad, frontal corners rounded, rather prominent over antennal bases; frontal disk depressed in common with epistoma, frontal stria recurved dorsad within depression, usually complete; sides of epistoma variably ridged, carinate and/or striate; labrum broad, apical margin generally carinate, truncate to weakly emarginate; mandibles with small or no basal teeth on incisor edge; submentum flat to slightly depressed, sparsely setose; mentum about half as long as wide, arcuately narrowed anteriorly, apical margin acutely emarginate; cardo smooth and glabrous, stipes with few setae along lateral margin; ultimate palpomeres fusiform; antennal scape elongate, curved, weakly carinate along anterior margin; funicle weakly widened beyond 5^th^ antennomere, 8^th^ antennomere slightly shorter than preceding; antennal club elongate, widest just beyond midpoint, tomentose, with interrupted basal annulus near midpoint and complete annulus between midpoint and apex, slightly enlarged basad at middle, particularly on dorsal surface. ***Pronotum*:** pronotal sides weakly arcuate, convergent to anterior corners, slightly to distinctly explanate at sides, marginal stria usually complete along lateral and apical margins, submarginal stria present very close to sides, absent across front, weakly crenulate; pronotum with pair of gland openings very close to anterior margin behind eye and glands variably displaced posterad onto pronotal disk, usually multiplied along a visible track bearing up to 5 distinct openings along its length; pronotal disk with secondary punctures, when present, strongly concentrated across basal half. ***Elytra*:** elytral disk weakly to moderately convex, all striae coarsely impressed, each stria comprising two alternating series of interconnected punctures, appearing chain-like at their most dense; epipleuron usually with one complete marginal stria, with fragments of a second in posterior half or not, inner subhumeral stria usually complete, dorsal striae 1-4 complete, 5^th^ and sutural striae rarely obsolete basally, bases of 4^th^ or 5^th^ and sutural striae rarely connected by weak basal arch; ground punctation of elytral disk fine to coarse, with at least a few coarse secondary punctures usually present in most interstriae. ***Prosternum*:** prosternal keel generally distinctly, subacutely emarginate at base, with complete carinal striae usually united in narrow anterior arch, short secondary lateral strioles frequently present between carinal striae and procoxae; lateral prosternal striae present, divergent in front of coxae; prosternal lobe one-half to two-thirds length of keel, apically rounded to subtruncate, with marginal stria present at middle, variably obsolete at sides. ***Mesoventrite*:** mesoventrite subacutely produced at middle, with complete marginal stria smooth to crenulate; mesometaventral stria crenulate, usually strongly arched to angulate anterad onto middle of mesoventral disk, disk frequently with sparse secondary punctures. ***Metaventrite*:** metaventral disk with coarse secondary punctures usually over most of surface, postmesocoxal stria present, recurved anterad to mesepimeron or ending freely posterolaterad coxa, lateral metaventral stria sinuate, extending from inner corner of mesocoxa toward middle of metacoxa, frequently abbreviated apically; coarse punctures of metepisternum may coalesce into vague to distinct striae. ***Abdomen*:** ventrites mostly coarsely punctate, punctures tending to coalesce into striae along apical margins of ventrites 2-4; 1^st^ ventrite with poorly developed lateral stria along inner edge of metacoxa, abbreviated or curving laterad behind coxa; propygidium transverse, 2-3× as wide as long, with single gland opening on each side, often borne on weak convexity behind anterolateral corner, sometimes associated with weak oblique striole, disk generally sparsely covered with shallow secondary punctures, punctures often sparser in apical half; pygidium more or less equilateral, apex rounded to weakly subacute, gland openings generally present near lateral margin one-fifth to one-fourth from base, these often associated with lateral marginal pygidial striae; marginal stria when present rarely complete around apex; pygidial disk variably punctate. ***Legs*:** all trochanters bearing single long seta; profemur rather dilated at middle, narrowed, slightly emarginate at inner apex, protibia generally rounded, strongly spinose, tibial margin only rarely emarginate between to form marginal teeth; protibial spurs present but generally weak; protarsus of both sexes with strongly spatulate setae; meso- and metafemora weakly dilated basad middle, with complete posterior marginal stria; meso- and metatibiae similar, weakly widened to apex, with series of long, rather thin marginal spines; meso- and metatarsi with 4 ventral spines along apical margins only. ***Male genitalia*:** accessory sclerites vestigial or absent; T8 with sides evenly tapered to subtruncate apex, basal membrane attachment line generally tangential to deep, rounded basal emargination, ventrolateral apodemes produced most strongly at base, narrowed apically, separated by about one-third T8 width beneath; S8 short along midline, with longer divergent lateral guides bearing several strong setae along apicoventral margin, with weak membraneous velum across entire apex; T9 with ventrolateral apodemes not strongly tapered, inner apices subquadrate beneath, T9 apices subacute to obliquely subtruncate; T10 entire, may be emarginate basally and/or apically; S9 rather short, with broad truncate to emarginate base, head variably subquadrate, apically emarginate, with apical flanges separate; aedeagus rather broad, flattened, sides rounded, apical division conspicuous, apices often distinctly separate, medioventral process strong, produced beneath near midpoint; basal piece one-fourth to one-third tegmen length, with distinct medioventral tooth; median lobe short, proximal arms strongly narrowed basally. ***Female genitalia*:** T8 forming single broad plate; S8 forming separate median and lateral plates, basal baculi thin, narrowly attached to lateral sclerites, convergent proximally; S9 slightly elongate, articulated with strap-shaped extension from apex of S8; T10 weakly sclerotized; valvifers paddle-shaped, paddles rather short, about one-third valvifer length; coxites 2.5–3× as long as wide, tridentate, with weak inner tooth, prominent median tooth, and intermediate lateral tooth; gonostyle nearly as long as median tooth, setose; bursa copulatrix membranous, lacking sclerites, weakly expanded; spermatheca weakly sclerotized, globose, borne on long thin stalk inserted at base of common oviduct, with elongate, spiraled spermathecal gland attached near its midpoint.

#### Diagnosis.

**Figure 18.**
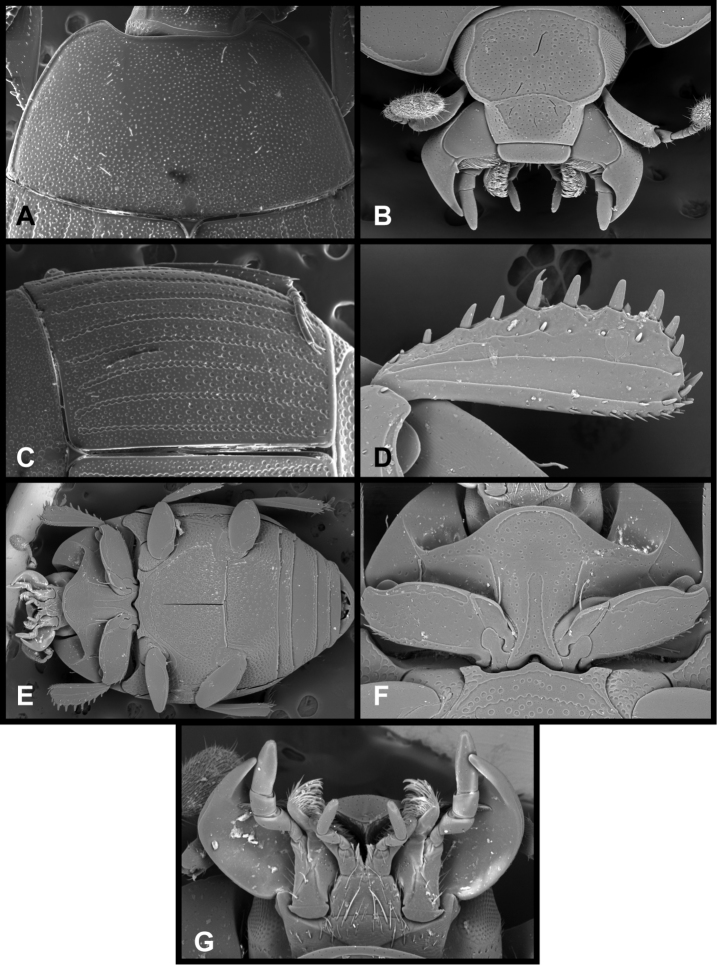
*Crenulister umbrosus*, SEMs showing generic characters. **A** Pronotum **B** Head, anterior view **C** Right elytron **D** Protibia, posterior view **E** Ventral habitus **F** Prosternum and mesoventrite **G** Head, ventral view.

**Figure 19.**
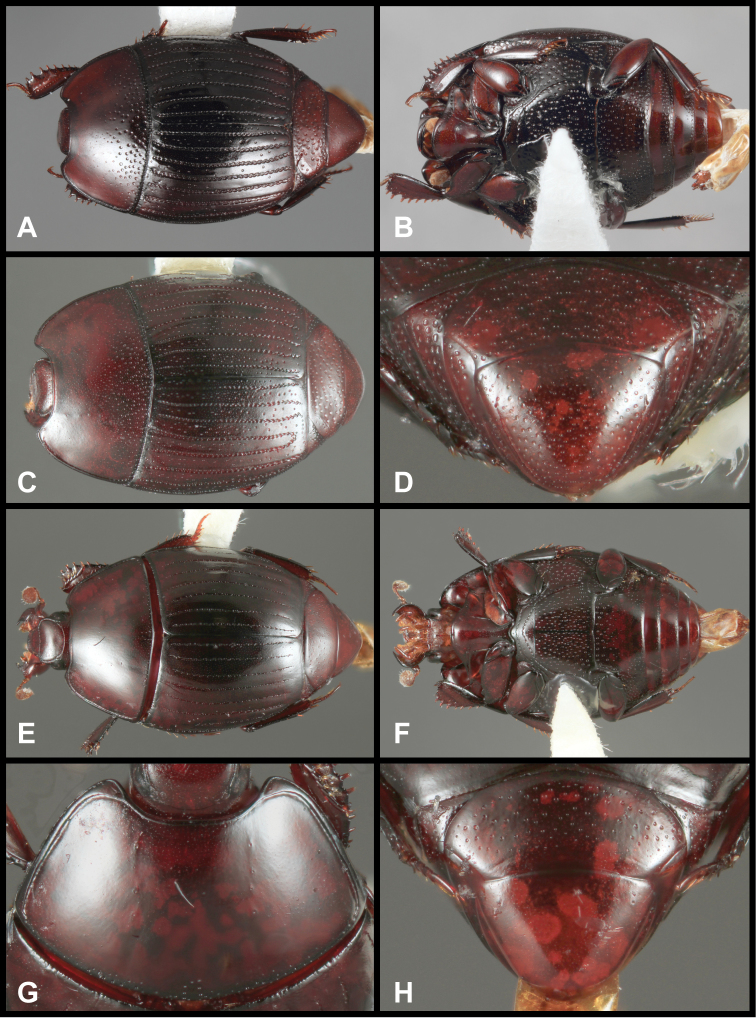
*Crenulister* spp. **A** *Crenulister grossus*, dorsal habitus **B** *Crenulister grossus*, ventral habitus **C** *Crenulister spinipes*, dorsal habitus **D** *Crenulister spinipes*, pygidia, posterior view **E** *Crenulister seriatus*, dorsal habitus **F** *Crenulister seriatus*, ventral habitus **G** *Crenulister seriatus*, pronotum **H** *Crenulister seriatus*, pygidia, posterior view.

**Figure 20.**
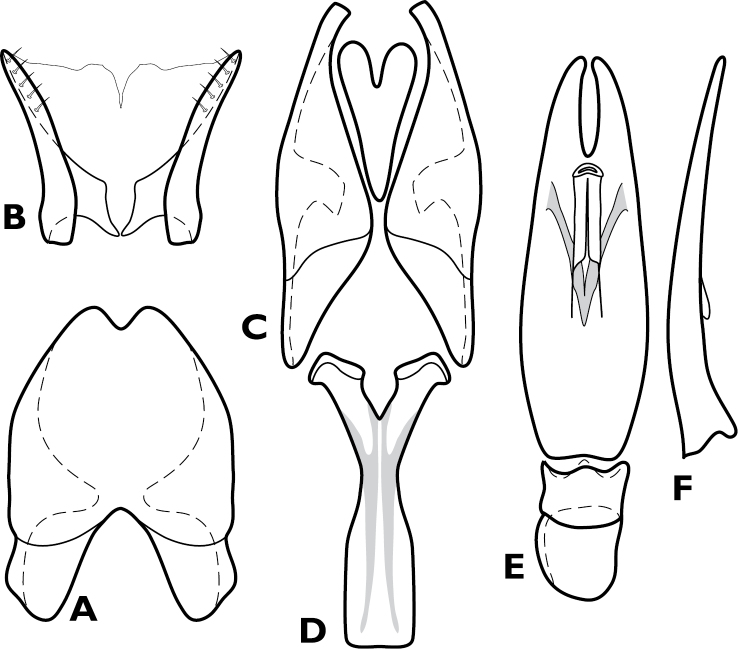
*Crenulister grossus*, male genitalia. **A** 8^th^ tergite **B** 8^th^ sternite **C** 9^th^ and 10^th^ tergites **D** 9^th^ sternite **E** Aedeagus, dorsal view **F** Aedeagus, lateral view.

In most respects this genus is highly distinctive. However, many of the distinguishing characters vary considerably within the group, in addition to appearing in unrelated species, and it is only through a combination of external characters and male genitalia can an unambiguous diagnosis be provided. For most species, the pattern of pronotal punctation, with coarse punctures restricted to the basal half of the pronotal disk (Figs 18A, 19A, C, 22A, 23A), is distinctive, and this in combination with coarsely impressed elytral striae (Fig. 18C) and sparse secondary punctures in the elytral interstriae will separate most species. The longitudinal tracks of multiplied pronotal gland openings (Fig. 19G) are also very unusual, and will put most species here easily. If the above are true, and the protarsi have spatulate setae, the diagnosis is unambiguous. Finally, the male genitalia of *Crenulister* also exhibit several distinctive characteristics, in particular the rather broad, flat aedeagus, with strong, acute ventromedial process (see Fig. 20E), frequently with the tegmen apices separated, the few strong setae at the apex of the 8^th^ sternite (Fig. 20B), and the broad, medially subquadrate ventrolateral apodeme of the 9^th^ tergite (Fig. 20C). A single (undescribed) species in a distantly related group shares all of these external characters, and represents an amazingly similar overall body form. The only good characters to distinguish it are its lack of spatulate protarsal setae, and its entirely different male genitalia, with its aedeagus strongly narrowed and hooked apically, completely unlike the short, broad and flattened aedeagus of *Crenulister*.

Phylogenetically, *Crenulister* emerges from within a diverse group of mostly undescribed taxa that we loosely term the ‘scutellar impression group’, particularly a small subgroup related to the named species *Phelister blairi* Hinton.

#### Etymology.

The genus name refers to the ubiquitously crenulate elytral striae found in the species of this genus, in conjunction with the common histerid ending -ister. The gender of the genus is masculine.

#### Key to species of *Crenulister*

**Table:** 

1	5^th^ and sutural striae obsolete in basal third	*Crenulister simplex*
–	5^th^ and usually sutural striae complete to base of elytra, at least as basal fragments	2
2	Inner subhumeral stria obsolete in basal fourth	*Crenulister seriatus*
–	Inner subhumeral stria reaching base of elytron	3
3	Punctures of metepisternum coalescing into distinct longitudinal stria half or more the length of the sclerite; marginal pygidial stria generally present, if substantially fragmented	4
–	Punctures of metepisternum discrete, or at most one or two coalescing into very short striole; marginal pygidial stria generally absent	6
4	Darker species; pygidial punctures denser near pygidial apex; epipleuron generally with second marginal stria well developed in posterior half	5
–	More rufescent species; pygidial secondary punctures less dense toward apex, with few secondary punctures along midline in general; epipleuron with only a single, complete epipleural stria	*Crenulister impar*
5	Larger species; prescutellar impression obsolete; marginal pygidial stria rather well impressed, may be interrupted apically, but rarely fragmented	*Crenulister spinipes*
–	Smaller species; small prescutellar impression evident among basal pronotal punctures, usually outlined by several punctures; marginal pygidial stria less regular, fragmented in apical third	*Crenulister umbrosus*
6	Pronotal disk very coarsely punctate in most of basal half, secondary punctures reaching midline at least in the middle; elytral striae deeply and coarsely impressed	*Crenulister grossus*
–	Pronotal disk with secondary punctures smaller and sparser, more or less restricted to basal third; elytral striae varied, but generally less strongly impressed	7
7	Larger (>3mm PE length), dark rufescent species, with moderately coarse punctures restricted to basal one-fourth of pronotum	*Crenulister paucitans*
–	Smaller (<2.5mm), more pale rufescent species, with secondary pronotal punctures smaller, sparser, and not as discretely limited to posterior one-fourth of pronotal disk	8
8	Outer protibial margin with emarginations between spines deep and distinct, nearly or fully as deep as the marginal spines are long; sutural stria complete to base	*Crenulister dentatus*
–	Outer protibial margin with emarginations between spines shallow, much shallower than length of marginal spines; sutural stria weakened to obsolete at base	*Crenulister explanatus*

### Crenulister grossus sp. n.

http://zoobank.org/6815E9BF-4D58-4FDB-B838-BED962A0C02C

http://species-id.net/wiki/Crenulister_grossus

**Map 4.**
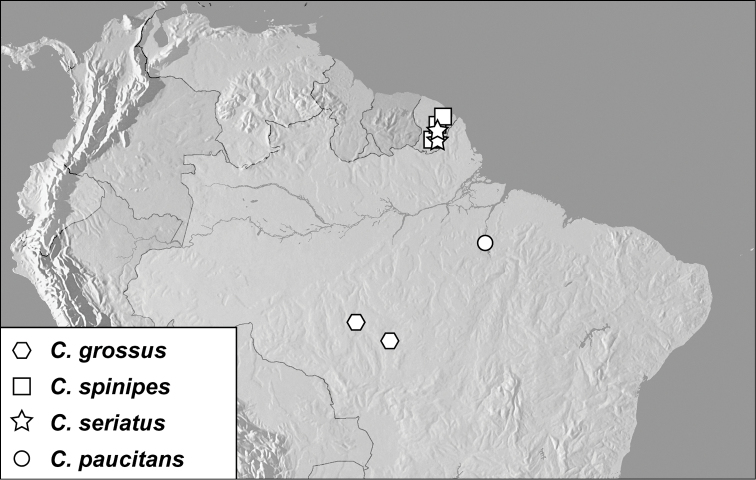
Specimen records of *Crenulister* spp.

Figs 19A–B , 20 , Map 4

#### Type locality.

BRAZIL: Mato Grosso, Fazenda São Nicolau [9.8386°S, 58.2508°W].

#### Type material.

**Holotype male:** “**BRASIL:** **Mato Grosso:** Mpio Cotriguaçu, Fazenda São Nicolau, Matinha. 9°50.3'S, 58°15.05'W. Flight intercept Oct 2009. F.Z.Vaz-de-Mello” / “Caterino/Tishechkin Exosternini Voucher EXO-03022” (CEMT). **Paratypes** (6): 1 same data as type, 2: same locality as type, but xii.2010; 3: **Mato Grosso:** Mpio Cláudia, 11°24.5'S, 55°19.5'W, FIT, 17–27.x.2010, A.F.Oliveira (CEMT, UFPR, FMNH, MSCC).

#### Diagnostic description.

Length: 2.6–3.2 mm, width: 2.4–2.8 mm; as for generic description, with the following diagnostic characters: body rufopiceous, elongate ovoid, moderately convex; frontal stria fine but complete across middle, frontal disk rather strongly depressed, with a few sparse secondary punctures, mostly toward vertex; epistoma with oblique lateral ridges delimiting median depression, but not striate; labrum about 5× wider than long, apical margin shallowly, broadly emarginate; pronotum with gland opening track extending posterad just beyond midpoint, with 3-4 openings along its length, substriate; pronotal sides moderately explanate along smooth, non-crenulate lateral submarginal stria; pronotal disk with numerous, large secondary punctures in basal third, most strongly concentrated and extending slightly further forward at middle, sides almost entirely smooth; prescutellar impression not evident; elytron with single, complete, crenulate epipleural stria, all dorsal striae complete, very coarsely impressed, appearing chain-like; elytral intervals very sparsely, irregularly punctate, most intervals with 4-6 punctures; prosternal keel with faint secondary striae alongside complete, anteriorly united carinal striae; prosternal lobe deflexed in apical half, with marginal stria present only at middle; mesoventrite with marginal stria weakly crenulate, mesometaventral stria more coarsely so and arched forward just beyond mesoventral midpoint; postmesocoxal stria recurved anterad around mesocoxa but ending short of mesepimeron; lateral metaventral stria crenulate, reaching middle of metacoxa, recurved mediad apically in some individuals; mesoventrite entirely and more or less uniformly coarsely punctate; metepisternal punctures independent, not forming a stria; punctures of 1^st^ abdominal ventrite mostly uniform, only transversely elongate along posterior margin; marginal punctures of ventrites 2–4 similarly elongate, but mostly separate, not or only intermittently coalescing into marginal striae; protibia 7-spined, with marginal dentation very weakly developed; meso- and metatibiae with 4-5 spines each, mainly in apical half; propygidium with secondary punctures shallow, very sparse, separated by 1-3× their diameters, densest in basal half; propygidial gland opening associated with very weak oblique striole in anterior corners; pygidial punctation weak, punctures much smaller and sparser than those of propygidium, more or less uniformly separated by about 4× their diameters throughout; pygidial gland openings evident at sides about one-fourth from base, marginal striae absent. Male (Fig. 20): accessory sclerites absent; T8 with ventrolateral apodemes strongly narrowed beneath; S8 with halves meeting only at basal corner, inner margins short and strongly divergent, with about 5 strong setae toward apex; T9 with apices obliquely truncate; T10 apically emarginate; S9 truncate, quadrate at base, apex narrowly and rather deeply emarginate; tegmen widest in basal third, narrowed to apex, apices slightly separated, medioventral process produced beneath about one-fourth from base; median lobe about one-third tegmen length, basal piece short, about one-fourth tegmen length.

#### Remarks.

This is the largest species of *Crenulister*, and between its size (Fig. 19A–B), coarse punctation, and very coarsely impressed elytral striae, one of the easiest to recognize.

#### Etymology.

This species name refers to its relatively large size, and secondarily its occurrence in Mato Grosso, Brazil.

### Crenulister spinipes sp. n.

http://zoobank.org/B93E12E9-34CC-4548-80C7-4A68545CCF94

http://species-id.net/wiki/Crenulister_spinipes

Figs 19C–D , Map 4

#### Type locality.

FRENCH GUIANA: Réserve des Nouragues [4.038°N, 52.673°W].

#### Type material.

**Holotype female:** “**GUYANE FRANÇAISE:** Régina, Réserve des Nouragues, 4°2.27'N, 52°40.35'W, Piége d’interception, 28 Jan 2010. SEAG leg.” / “Caterino/Tishechkin Exosternini Voucher EXO-00181” (MNHN). **Paratypes** (2): 1: Belvèdére de Saül, point de vue, 3°1'22"N, 53°12'34"W, 4.i.2011, FIT, SEAG (CHND); 1: Montagne des Chevaux, 4°43'N, 52°24'W, 26.xii.2008, FIT, SEAG (FMNH)

#### Diagnostic description.

Length: 2.4–2.6 mm, width: 2.2–2.4 mm; as for generic description with the following diagnostic characters: body rufobrunneus, elongate ovoid, moderately convex; frontal stria fine but complete across middle, frontal disk rather strongly depressed, with sparse secondary punctures rather evenly distributed on frons and epistoma; epistoma with oblique lateral ridges delimiting median depression bearing faint traces of striae basally; labrum about 4× wider than long, apical margin shallowly, broadly emarginate; pronotum with gland opening track extending posterad just beyond midpoint, with 3-4 openings along its length; pronotal sides moderately explanate along very weakly crenulate lateral submarginal stria; pronotal disk with numerous, large secondary punctures in basal third, most strongly concentrated at middle, sides largely smooth; prescutellar impression not evident; elytron with one complete, crenulate epipleural stria and second incomplete stria closer to margin in posterior half, all dorsal striae complete, moderately coarsely impressed, appearing chain-like; elytral intervals sparsely, irregularly punctate, most intervals with 12–18 punctures; prosternal keel with complete, narrowly anteriorly united carinal striae; prosternal lobe with marginal stria present only at middle; mesoventrite with marginal stria weakly crenulate, mesometaventral stria similarly so, subangulately arched forward just beyond mesoventral midpoint; postmesocoxal stria slightly recurved anterad around mesocoxa but ending short of mesepimeron; lateral metaventral stria crenulate, reaching middle of metacoxa; mesoventrite entirely and more or less uniformly coarsely punctate; metepisternal punctures coalescing into a short longitudinal stria; punctures of 1^st^ abdominal ventrite mostly uniform, slightly obliquely elongate posterad metacoxa, transversely elongate along posterior margin, intermittently coalesced into marginal strioles, as are those of ventrites 2–4; protibia 7–8-spined, with marginal dentation very weakly developed; meso- and metatibiae with 4–5 spines each, mainly in apical half; propygidium with secondary punctures shallow, sparse, separated by 1–2× their diameters throughout; propygidial gland opening associated with weak oblique striole in anterior corners; pygidial punctation sparse, punctures slightly smaller than those of propygidium, more or less uniformly separated by 2–3× their diameters throughout; pygidial gland openings evident at sides about one-fourth from base, marginal striae present along sides, broadly interrupted at apex. Male: not known.

#### Remarks.

This species and *Crenulister grossus* are very similar and evidently closely related. The smaller size, less coarsely impressed elytral striae (Fig. 19C), and semistriate metepisternum, abdominal ventrites and propygidium (Fig. 19D) will distinguish this species readily. Although both are larger than average for the genus, *Crenulister grossus* is still markedly larger.

#### Etymology.

The name of this species refers to its conspicuously spinose protibiae.

### Crenulister seriatus sp. n.

http://zoobank.org/6A69B05A-9B17-48B4-B74E-D95D8B5770F7

http://species-id.net/wiki/Crenulister_seriatus

**Figure 21.**
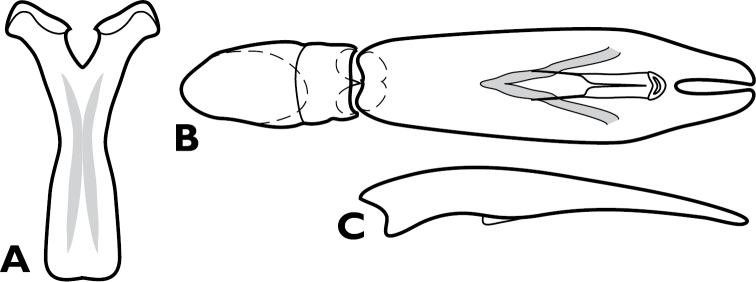
*Crenulister seriatus*, male genitalia. **A** 9^th^ sternite **B** Aedeagus, dorsal view **C** Aedeagus, lateral view.

Figs 19E–H , 21 , Map 4

#### Type locality.

FRENCH GUIANA: Belvèdére de Saül [3.01°N, 53.21°W].

#### Type material.

**Holotype male:** “**GUYANE FRANÇAISE:** Bélvédère de Saúl, point de vue. 3°1'22"N, 53°12'34"W. Piège vitre, 20.xii.2010. SEAG leg.” / “Caterino/Tishechkin Exosternini Voucher EXO-01768” (MNHN). **Paratypes** (5): 4: same locality as type, 7.i.2011, 24.i.2011, 31.i.2011, and 2.ix.2011 (CHND, FMNH, MSCC); 1: Régina, Réserve des Nouragues, 4°2.27'N, 52°40.35'W, 3.ix.2009, FIT, SEAG (CHND).

#### Diagnostic description.

Length: 2.7–3.0 mm, width: 2.2–2.4 mm; as for generic description with the following diagnostic characters: body dark rufobrunneus, elongate oval, weakly convex; frontal stria fine, complete, frontal disk shallowly depressed, with few or no secondary punctures; epistoma with weak lateral ridges delimiting median depression bearing weak basal strioles; labrum about 4× wider than long, apical margin weakly outwardly arcuate; pronotum with gland opening track extending posterad behind middle, with 4 more or less evenly spaced openings; pronotal sides weakly depressed along smooth, raised lateral submarginal stria; pronotal disk with only a single series secondary punctures along basal margin, with a few encircling weak prescutellar impression, sides impunctate; elytron with one complete, crenulate epipleural stria and second, incomplete stria closer to margin in posterior half, inner subhumeral stria variably obsolete in about basal third, all other dorsal striae complete, moderately coarsely impressed, appearing chain-like; interstriae almost entirely lacking secondary punctures; prosternal keel with complete carinal striae slightly divergent anteriorly, not connected, faint secondary carinal stria present between carinal stria and procoxa; prosternal lobe deflexed in anterior half, with marginal stria present only at middle; mesoventrite with marginal stria weakly crenulate, mesometaventral stria crenulate, subangulately arched forward just beyond mesoventral midpoint; postmesocoxal stria slightly recurved anterad around mesocoxa, nearly reaching mesepimeron; lateral metaventral stria not crenulate, reaching inner third of metacoxa; metaventrite with secondary punctation largely uniform, but median punctures slightly smaller than those at sides; metepisternal punctures vaguely coalesced into a short stria; lateral stria of 1^st^ abdominal ventrite well impressed along inner edge of metacoxa, with a separate transverse striole behind metacoxa; punctures of 1^st^ abdominal ventrite limited to anterior third of median part of disk, with few oblique punctures posterad metacoxa; posterior margins of ventrites 1-4 very narrowly striatopunctate; protibia 7-8-spined, with marginal dentation weakly developed; mesotibia with 6-8 spines along margin, metatibia with fewer spines, present mainly along apical half; propygidium with secondary punctures quite small, sparse, separated by 2-4× their diameters, becoming obsolete in apical fourth; propygidial disk lacking anterolateral strioles; pygidium lacking secondary punctures; pygidial gland openings distinct about one-sixth from anterior margin, equidistant from lateral margin; pygidial margin lacking striae. Male (T8, S8, T9 as in *Crenulister grossus*; see Fig. 20): accessory sclerites absent; T8 with ventrolateral apodemes strongly narrowed beneath; S8 with halves meeting only at basal corner, inner margins short and strongly divergent, with about 7 strong setae toward apex; T9 with apices narrowly obliquely truncate; T10 desclerotized along much of midline, only undivided at middle; S9 short, broad, widened to weakly emarginate base, apex broadly emarginate (Fig. 21A); tegmen (Fig. 21B–C) with sides weakly rounded, widening to just beyond midpoint, narrowed to apex, apices slightly separated, medioventral process produced beneath about one-third from base; median lobe about one-third tegmen length, basal piece just over one-third tegmen length.

#### Remarks.

This species is quite distinguishable by its relative lack of secondary punctures (Fig. 19E). Those of the pronotum occur only along the basal margin; those of the interstriae are essentially lacking; the 1^st^ abdominal ventrite is punctate only in the anterior third; and the pygidium (Fig. 19H) is devoid of secondary punctures. Among the larger, darker species, these features readily diagnose it.

#### Etymology.

This species’ name refers to its particularly conspicuous series of pronotal gland openings, against a largely impunctate background.

### Crenulister paucitans sp. n.

http://zoobank.org/CDD37AA2-5C9E-485F-BF2D-6C45BF1BE97A

http://species-id.net/wiki/Crenulister_paucitans

Fig. 22 , Map 4

#### Type locality.

BRAZIL: Pará: Tucuruí [3.75°S, 49.67°W].

#### Type material.

**Holotype female:** “**BRASIL:** **Pará:** Tucuruí, 3°45'S, 49°40'W. Piège d’interception. 27.x-9.xi. 1985” / “Caterino/Tishechkin Exosternini Voucher EXO-00180” (UFPR).

#### Diagnostic description.

Length: 3.1 mm, width: 2.6 mm; as for generic description with the following diagnostic characters: body rufobrunneus, elongate ovoid, moderately convex; frontal stria fine, very narrowly interrupted at middle, frontal disk moderately depressed, with very sparse secondary punctures barely larger than background punctation; epistoma with weak lateral ridges delimiting median depression; labrum about 4× wider than long, apical margin more or less straight across; pronotum with gland opening track extending posterad to about midpoint, with 3 openings lying within weak striole in its posterior half; pronotal sides explanate along crenulate lateral submarginal stria; pronotal disk with secondary punctures restricted to basal margin, the smallest barely extending beyond the basal fourth, sides largely smooth; prescutellar impression not evident; elytron with one complete, crenulate epipleural stria and posterior fragments of second incomplete stria closer to margin, all dorsal striae complete, moderately coarsely impressed, appearing chain-like; elytral intervals very sparsely, irregularly punctate, most intervals with 8-12 small punctures; prosternal keel with complete carinal striae slightly separated anteriorly; prosternal lobe slightly deflexed, with marginal stria present only at middle; mesoventrite with marginal stria smooth, mesometaventral stria crenulate, subangulately arched forward just to about mesoventral midpoint; postmesocoxal stria slightly recurved anterad around mesocoxa, but ending short of mesepimeron; lateral metaventral stria weakly crenulate, reaching middle of metacoxa; metaventrite with secondary punctures larger and denser in posterior half; metepisternal punctures not coalesced into a short stria; lateral stria of 1^st^ abdominal ventrite well impressed along inner edge of metacoxa, with a separate transverse striole behind metacoxa; punctures of 1^st^ abdominal ventrite rather small, finer and sparser posterad, slightly obliquely elongate posterad metacoxa, transversely elongate along posterior margin, intermittently coalesced into marginal strioles, as are those of ventrites 2-4; protibia 7-8-spined, with marginal dentation weakly developed; mesotibia with 6 spines along margin, metatibia with 4 spines, mainly in apical half; propygidium with secondary punctures shallow, sparse, separated by 1-3× their diameters throughout; propygidial disk lacking anterolateral strioles; pygidial punctation very sparse and fine, almost obsolete along midline; pygidial gland openings slightly tuberculate, evident near sides about one-fifth from base; pygidial margin lacking striae. Male: not known.

#### Remarks.

**Figure 22.**
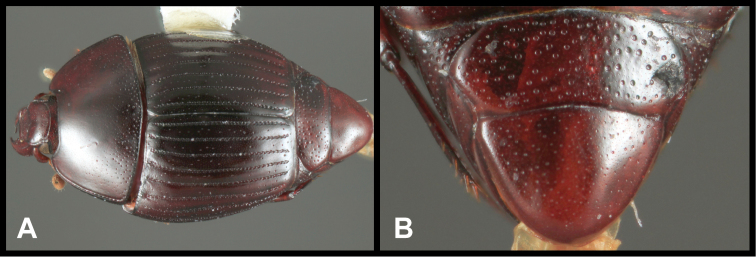
*Crenulister paucitans*. **A** Dorsal habitus **B** Pygidium, posterior view.

Given the existence of only a single specimen, it seems likely that many apparently distinct features may become less so in light of individual variation. However, this species can generally be distinguished by the strong limitation of pronotal punctures to the basal fourth (Fig. 22A), the association of the pronotal gland openings with a distinct longitudinal striole, the diminished punctation in the anterior half of the metaventral disk, and the lack of marginal pygidial stria (Fig. 22B).

#### Etymology.

The name of this species refers to the relative paucity of punctures in the elytral interstriae.

### Crenulister umbrosus sp. n.

http://zoobank.org/BC4A74B9-1E52-4549-825A-F4B591B52FB7

http://species-id.net/wiki/Crenulister_umbrosus

**Map 5.**
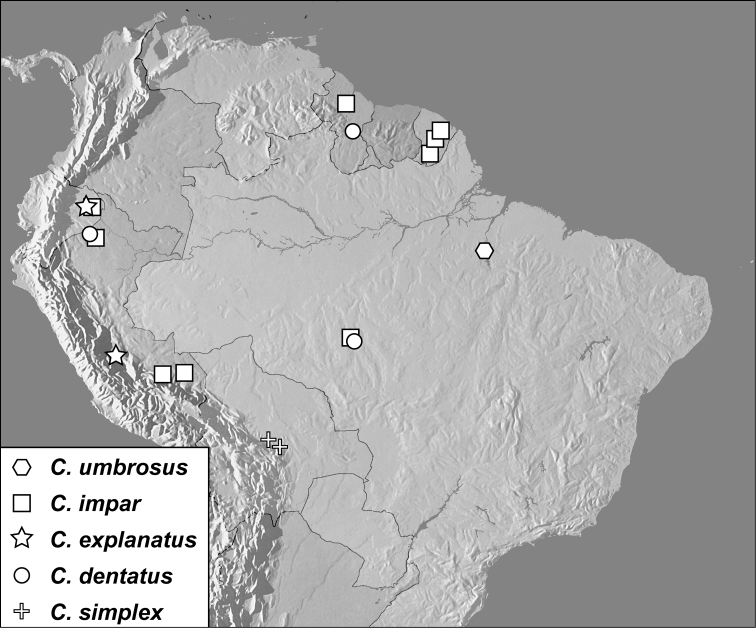
Specimen records of *Crenulister* spp.

Figs 18A–G , 25E–G , Map 5

#### Type locality.

BRAZIL: Pará: Tucuruí [3.75°S, 49.67°W].

#### Type material.

**Holotype male:** “**BRASIL:** **Pará**, Tucurui, 3°45'S, 49°40'W. Piège d’interception. IV.1985” / “Caterino/Tishechkin Exosternini Voucher EXO-03005” (UFPR). **Paratypes** (3): same locality as type, 2: 10-29.vii.1985 (CHND, MSCC, FMNH), 1: 9-17.xii.1985 (CHND).

#### Other material.

Same locality as type, 9–17.xii.1985 (CHND).

#### Diagnostic description.

Length: 1.9–2.0 mm, width: 1.7–1.8 mm; as for generic description with the following diagnostic characters: body rufopiceous, elongate ovoid, slightly more parallel-sided than most, subdepressed; frontal stria fine, complete, frontal disk moderately depressed, with fine, dense punctation consisting of ground punctation and barely larger secondary punctation; epistoma with lateral ridges delimiting median depression bearing weak fragments of lateral striae; labrum about 4× wider than long, apical margin weakly emarginate; pronotum with gland opening track nearly reaching midline, with 3-4 openings lying within the impunctate track; pronotal sides with weakly crenulate, complete lateral submarginal stria; secondary punctures of pronotal disk rather small, mostly restricted to basal fourth; weak prescutellar impression present; elytron with complete, crenulate epipleural stria and secondary stria in posterior half; all dorsal striae complete, coarsely impressed; elytral interstriae irregularly but distinctly punctate, intervals with 20–25 coarse punctures; prosternal keel deeply emarginate at base, with carinal striae slightly abbreviated anteriorly, united; prosternal lobe slightly deflexed, marginal stria present only at middle; mesoventrite with marginal stria weakly crenulate, mesometaventral stria crenulate, arched forward to basal third of mesoventral disk; postmesocoxal stria recurved anterad around mesocoxa, ending short of mesepimeron; lateral metaventral stria rather weakly crenulate, ending short of metacoxa; metaventral disk with secondary punctures slightly smaller and sparser anteromedially; metepisternal punctures coalesced into a short stria; lateral stria of 1^st^ abdominal ventrite fragmentary along inner edge of metacoxa, curving laterad behind metacoxa; secondary punctures of median portion of 1^st^ abdominal ventrite smaller and sparser in posterior half, with slightly oblique punctures toward sides behind metacoxa; punctures along posterior margins of ventrites 1–2 transversely elongate but separate, those of ventrites 3–4 intermittently coalesced into marginal strioles; protibia 5–6-spined, with marginal dentation weakly developed; meso- and metatibia with thin, elongate spines, mainly along apical half of margin; propygidium with secondary punctures shallow but rather large, uniformly separated by 1–1.5× their diameters; propygidial gland openings borne on faint tubercles in anterolateral corners; propygidial strioles absent; pygidium with secondary punctures more conspicuous at sides and apex; pygidial gland openings present near sides about one-fourth from base; pygidial margin with fragmentary striae along apical two-thirds, not complete around apex. Male (Figs 25E-G): accessory sclerites absent; T8 with ventrolateral apodemes strongly narrowed beneath; S8 with halves meeting along short inner margins apices moderately strongly divergent, with about 5 strong setae; T9 with apices narrow, obliquely truncate; T10 weakly desclerotized along midline; S9 slightly widened to weakly emarginate base, apex rather deeply emarginate; tegmen with sides very weakly rounded, widest toward apex, apices rather narrow and slightly separated, medioventral process produced beneath about one-fourth from base; median lobe nearly one-half tegmen length, basal piece about one-third tegmen length.

#### Remarks.

This species is the darkest of the smaller species. Its coloration in combination with complete elytral striation, conspicuous interstrial punctation (Fig. 18C), and fragmentary marginal pygidial stria will fairly readily distinguish it from other species in the genus. The single specimen excluded from the type series is slightly larger and more coarsely punctate, especially ventrally, than the other specimens, despite being collected in the same locality (and even same dates).

#### Etymology.

This species’ name refers to its relatively dark coloration.

### Crenulister impar sp. n.

http://zoobank.org/2DFA0072-A31C-48B5-8871-DCC23E0B38FB

http://species-id.net/wiki/Crenulister_impar

**Figure 23.**
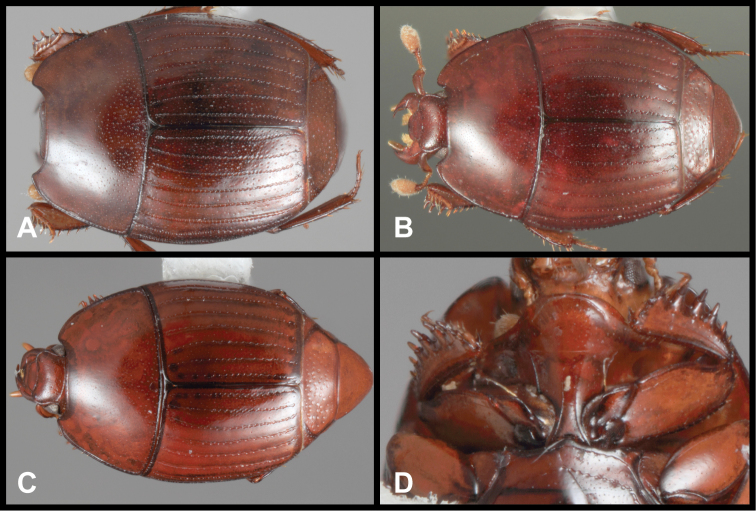
*Crenulister* spp. **A** *Crenulister impar*, dorsal habitus **B** *Crenulister explanatus*, dorsal habitus **C** *Crenulister dentatus*, dorsal habitus **D** *Crenulister dentatus*, prosternum and protibiae.

**Figure 24.**
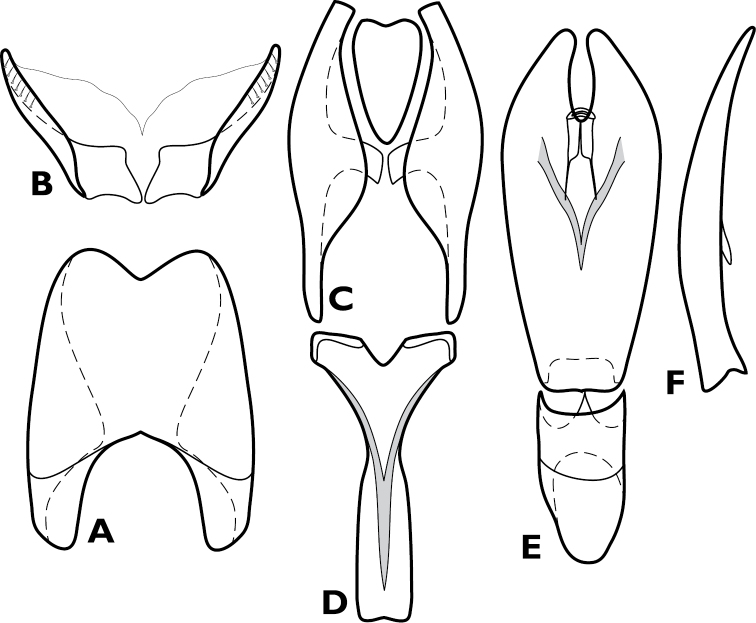
*Crenulister impar*, male genitalia. **A** 8^th^ tergite **B** 8^th^ sternite **C** 9^th^ and 10^th^ tergites **D** 9^th^ sternite **E** Aedeagus, dorsal view **F** Aedeagus, lateral view.

Figs 23A , 24 , Map 5

#### Type locality.

FRENCH GUIANA: Belvèdére de Saül [3.01°N, 53.21°W].

#### Type material.

**Holotype male:** “**GUYANE FRANÇAISE:** Bélvédère de Saúl, point de vue. 3°1'22"N, 53°12'34"W. Piège vitre, 2.ix.2011. SEAG leg.” / “Caterino/Tishechkin Exosternini Voucher EXO-03016” (MNHN). **Paratypes** (12): 1: same locality as type, 20.xii.2010 (CHND); 4: **FRENCH GUIANA:** Rés. Natur. des Nouragues, Camp Inselberg, 4°05'N, 52°41'W, 30.ix.2010, FIT, SEAG (MNHN, CHND, FMNH, MSCC), 1: 20.viii.2010 (LSAM), 1: 22.ix.2010 (CHND), 1: 9.xi.2010 (CHND), 1: 25.i.2011 (CHND), 1: 8.x.2010 (CHND); 1: Matoury, 41.5 km SSW on Hwy N2, 4°37'22"N, 52°22'35"W, 50m, 26-28.v.1997, J. Ashe & R. Brooks, FIT (SEMC). 1: **GUYANA:** **Cuyuni-Mazaruni:** Kartabo, 24.ix.1920, W.M.Mann (USNM).

#### Other material.

1: **PERU:** **Madre de Dios:** Tambopata, Reserva Cuzco Amazonico, 15km NE Pto. Maldonado, 12°33'S, 69°03'W, 200m, 22.vi.1989, FIT, J. Ashe & R. Leschen (SEMC), 1: 24.vi.1989 (SEMC); 1: **Loreto:** 1.5km N Teniente Lopez, 2°35.66'S, 76°06.92'W, 210–240m, 20.vii.1993, FIT, R. Leschen (SEMC); 1: **Cusco:** Villa Carmen Field Station, 12.8925°S, 71.4192°W, 24-26.v.2011, FIT (SEMC). 1: **BRAZIL:** **Mato Grosso**, Cotriguaçu, Fazenda São Nicolau, Matinha, 9°50.3'S, 58°15.05'W, xii.2009, FIT, F.Z. Vaz-de-Mello (CEMT). 1: **ECUADOR:** **Orellana:** Tiputini Biodiversity Station, 0.6376°S, 76.1499°W, 4–9.vi.2011, FIT, M.S. Caterino & A.K. Tishechkin, DNA extraction voucher MSC-2129 (MSCC).

#### Diagnostic description.

Length: 1.8–2.2 mm, width: 1.6–2.0 mm; as for generic description with the following diagnostic characters: body rufescent to rufobrunneus, elongate ovoid, subdepressed; frontal stria fine, usually complete, rarely narrowly interrupted, frontal disk moderately depressed, with fine but relatively dense punctation consisting of ground punctation and barely larger secondary punctation; epistoma with lateral ridges delimiting median depression bearing weak lateral striae basally; labrum about 4× wider than long, apical margin weakly emarginate; pronotum with gland opening track reaching approximately to midline, with 3-4 openings along its length; pronotal sides with crenulate, slightly elevated lateral submarginal stria; pronotal disk with small secondary punctures conspicuous in basal half, as well as at sides, anteromedial portion of disk with only fine ground punctation; weak prescutellar impression present; elytron with one complete, crenulate epipleural stria rather distant from margin, especially posteriorly, all dorsal striae complete, shallowly but coarsely impressed; all elytral interstriae sparsely, irregularly punctate, with 18-30 secondary punctures, generally more in the 5^th^-sutural interval; prosternal keel deeply emarginate at base, carinal striae complete, narrowly united anteriorly; prosternal lobe slightly deflexed, marginal stria present only at middle; mesoventrite with marginal stria fine to weakly crenulate, mesometaventral stria crenulate, angulately arched forward to basal third of mesoventral disk; postmesocoxal stria recurved anterad around mesocoxa, ending short of mesepimeron; lateral metaventral stria crenulate, ending short of metacoxa; metaventral disk punctate throughout, secondary punctures slightly smaller and sparser anteromedially; metepisternal punctures coalesced into distinct stria; lateral stria of 1^st^ abdominal ventrite present along inner edge of metacoxa, curving laterad behind metacoxa, occasionally interrupted; secondary punctures of median portion of 1^st^ abdominal ventrite larger and denser in basal half, with slightly oblique punctures toward sides behind metacoxa; punctures along posterior margins of ventrites 1-4 transversely elongate, intermittently coalesced into marginal strioles; protibia ~6-7-spined, with marginal dentation weak; meso- and metatibia with 3-5 thin, elongate spines, mainly along apical half of margin; propygidium with secondary punctures shallow but rather large, fairly uniformly separated by about their diameters throughout; propygidial gland openings very faintly tuberculate in anterolateral corners, propygidial strioles absent; pygidium with sparse secondary punctation mainly along sides; pygidial gland openings evident near sides about one-fourth from base; pygidial margin with striae along most of apical two-thirds, usually interrupted at apex. Male (Fig. 24): accessory sclerites absent; T8 with ventrolateral apodemes strongly narrowed beneath; S8 short with halves meeting only at basal corner, inner margins short and strongly divergent, with 6-8 strong setae toward apex; T9 with apices obliquely truncate; T10 weakly emarginate at apex; S9 quadrate at base, apex emarginate; tegmen widest in apical third, apex more or less rounded, apices slightly separated, medioventral process produced beneath about one-third from base; median lobe short, basal piece about one-third tegmen length.

#### Remarks.

This species is somewhat difficult to characterize, in part because there's, relatively more material than for most, from a wider area, and individual variation is accordingly more evident. Among the smaller, more rufescent species of the genus, the complete elytral striae (Fig. 23A), presence of distinct secondary punctures on most of the basal half of the pronotal disk, the pronotal gland track reaching to the pronotal midpoint, and the presence of (usually) well developed marginal pygidial striae will generally distinguish it. The apically widened aedeagus (Fig. 24E) is distinctive, and consistent among males examined. However, we restrict the type locality to the Guianas due to some uncertainty about species assignment of all populations. Additional material from the southern and Andean parts of the range may reveal more consistent patterns of external variation and justify further splitting.

#### Etymology.

This species’ name refers to the ‘unequal’ distribution of pronotal punctures, distinctly increasing in density posterad.

### Crenulister explanatus sp. n.

http://zoobank.org/4E3D496A-4682-430E-873A-EE533BD83C3E

http://species-id.net/wiki/Crenulister_explanatus

**Figure 25.**
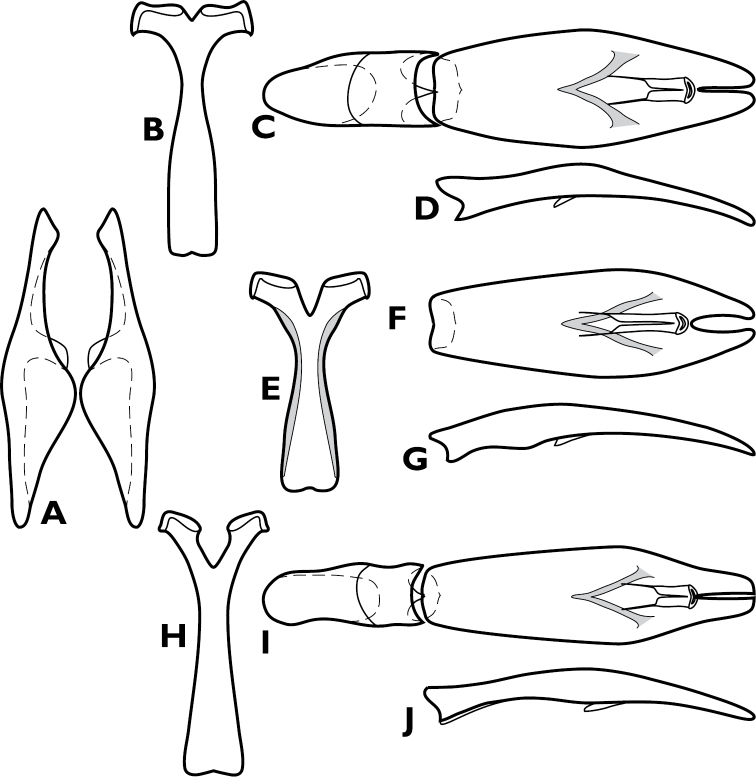
*Crenulister* spp., male genitalia. **A** *Crenulister dentatus*, 9^th^ tergite **B** *Crenulister explanatus*, 9^th^ sternite **C** *Crenulister explanatus*, aedeagus, dorsal view **D** *Crenulister explanatus*, aedeagus, lateral view **E** *Crenulister umbrosus*, 9^th^ sternite **F** *Crenulister umbrosus*, aedeagus, dorsal view **G** *Crenulister umbrosus*, aedeagus, lateral view **H** *Crenulister dentatus*, 9^th^ sternite **I** *Crenulister dentatus*, aedeagus, dorsal view **J** *Crenulister dentatus*, aedeagus, lateral view.

Figs 23B , 25B–D , Map 5

#### Type locality.

ECUADOR: Orellana, Yasuní Research Station [0.674°S, 76.398°W].

#### Type material.

**Holotype male:** “**ECUADOR:** Napo, mid.Rio Tiputini, Yasuní Res. Stn. 0°40.5'S, 76°24'W, FIT#1, 23-30 Jun 1999. AKT#032, C.Carlton & A.Tishechkin” / “LSAM0045737” (FMNH).

#### Other material.

1: same locality as type, 26.vii–4.viii.1999 (LSAM); 1: **PERU:** **Junin**, ~16km NW Satipo, Rio Venado, 11°11.677'S, 74°46.137'W, 1150m, 3-8.iii.2010, A.V. Petrov (LSAM).

#### Diagnostic description.

Length: 1.8–2.1 mm, width: 1.6–1.9 mm; as for generic description with the following diagnostic characters: body rufescent, elongate ovoid, subdepressed; frontal stria fine, complete, frontal disk moderately depressed, frontal stria fine, complete, disk with few rather conspicuous secondary punctures against fine background punctation; epistoma with rather well-developed lateral and anterior marginal striae; labrum about 4× wider than long, apical margin subtruncate; pronotum with single median gland opening about one-third from anterior margin; pronotal sides with fine, slightly raised lateral submarginal stria; secondary punctures of pronotal disk small, sparse, slightly larger and denser in basal third; prescutellar impression small but distinct; elytron with single, complete, crenulate epipleural stria; subhumeral and dorsal striae 1-4 complete, 4^th^ weakly arched to the sutural at base, 5^th^ stria barely abbreviated basally, sutural stria largely obsolete in basal fourth, may be represented by few serial punctures, all striae shallowly but coarsely impressed; inner elytral interstriae irregularly, sparsely punctate, with 8-10 punctures, fewer in outer intervals; prosternal keel acutely but not too deeply emarginate at base, with carinal striae narrowly united anteriorly; prosternal lobe weakly deflexed, marginal stria present only at middle; mesoventrite with marginal stria fine, mesometaventral stria crenulate, subangulate to basal third of mesoventrite; postmesocoxal stria recurved anterad around mesocoxa, reaching mesepimeron; lateral metaventral stria weakly crenulate, abbreviated in front of metacoxa; metaventral disk with secondary punctures smaller and sparser anteromedially; metepisternal punctures separate, not coalesced into a stria; lateral stria of 1^st^ abdominal ventrite weakly impressed along inner edge of metacoxa; secondary punctures of median portion of 1^st^ abdominal ventrite becoming obsolete in posterior half, with slightly oblique punctures toward sides behind metacoxa; punctures along posterior margins of ventrites 1-4 transversely elongate, intermittently coalesced into marginal strioles; protibia 5-6 spined, marginal dentation weakly developed; meso- and metatibia with 4-5 thin, elongate spines, mainly along apical half of margin; propygidium with secondary punctures shallow but rather large, uniformly separated by about half their diameters in basal half, sparser in posterior half; propygidial gland openings present in anterolateral corners; propygidial strioles absent; pygidium with few small secondary punctures, more conspicuous at sides; pygidial gland openings not evident; pygidial margin lacking striae. Male (Fig. 25B–D): accessory sclerites weakly sclerotized, present; T8 with ventrolateral apodemes rather broad beneath, narrowing to apex; S8 with halves meeting only at basal corner, inner margins short and strongly divergent, with about 3 strong setae toward apex; T9 with apices subacute; T10 apically entire; S9 weakly emarginate, quadrate at base, apex narrowly and shallowly emarginate; tegmen widest near midpoint, apices only very narrowly separated, medioventral process produced beneath about one-third from base; median lobe about one-third tegmen length, basal piece nearly half tegmen length.

#### Remarks.

This species is closely related to the following, with simple pronotal gland openings, the elytral interstrial punctures largely restricted to the inner intervals, the postmesocoxal stria reaching the mesepimeron, and the metepisternum nonstriate. This species does not have the protibial margin appearing as strongly dentate, and has the sutural stria basally obsolete. The three specimens we assign to this species in fact vary considerably in size, coloration, and sculpturing, and we exclude two of them (females) from the type series due to associated uncertainty, regardless the fact that one is from the same locality as the holotype.

#### Etymology.

This species’ name refers to the slightly explanate lateral pronotal margins (Fig. 23B).

### Crenulister dentatus sp. n.

http://zoobank.org/87F03327-D81B-4ED0-9539-943F66D08A2B

http://species-id.net/wiki/Crenulister_dentatus

Figs 23C–D , 25H–J , Map 5

#### Type locality.

GUYANA: Potaro-Siparuni (Region 8), Iwokrama Forest [4.2844°N, 58.5097°W].

#### Type material.

**Holotype male:** “GUYANA: Region 8, Iwokrama Forest, Kabocalli Field Stn., 60m, 4°17'4"N, 58°30'35"W, 3–5 JUN 2001 R. Brooks,Z.Falin GUY1BF01 146 ex: flight intercept trap” / “SM0573091 KUNHM-ENT [barcode label]” (SEMC).

#### Other material.

1: **BRAZIL:** **Mato Grosso:** Cotriguaçu, Fazenda São Nicolau, Prainha, 9°51.6'S, 58°12.9'W, x.2009 (CEMT); 1: **PERU:** **Loreto:** Campamento San Jacinto, 2°18'44.85"S, 75°51'46"W, 175-215m, 3-12.vii.1993, R. Leschen, FIT (SEMC).

#### Diagnostic description.

Length: 1.9–2.0 mm, width: 1.6–1.8 mm; as for generic description with the following diagnostic characters: body rufescent, elongate ovoid, subdepressed; frontal stria well impressed, complete, frontal disk moderately depressed, with few rather conspicuous secondary punctures against fine background punctation; epistoma with lateral ridges delimiting median depression bearing very weak fragments of lateral striae; labrum about 4× wider than long, apical margin weakly emarginate; pronotum with single median gland opening about one-third from anterior margin; pronotal sides with weakly crenulate, slightly raised lateral submarginal stria; secondary punctures of pronotal disk small, sparse, slightly larger and denser in basal half; weak prescutellar impression present; elytron with single complete crenulate epipleural stria; all dorsal striae complete, coarsely impressed; elytral interstriae irregularly, sparsely punctate, inner intervals with 8–10 punctures, fewer in outer intervals; prosternal keel deeply emarginate at base, with carinal striae slightly abbreviated, separate anteriorly; prosternal lobe deflexed, marginal stria present only at middle; mesoventrite with marginal stria weakly crenulate, mesometaventral stria crenulate, subangulate to near mesoventral midpoint; postmesocoxal stria recurved anterad around mesocoxa, reaching mesepimeron; lateral metaventral stria rather weakly crenulate, extending nearly to middle of metacoxa; metaventral disk with secondary punctures distinctly smaller and sparser anteromedially; metepisternal punctures separate, not coalesced into a stria; lateral stria of 1^st^ abdominal ventrite weakly impressed along inner edge of metacoxa; secondary punctures of median portion of 1^st^ abdominal ventrite smaller and sparser in posterior half, with slightly oblique punctures toward sides behind metacoxa; punctures along posterior margins of ventrites 1–2 transversely elongate but separate, those of ventrites 3–4 intermittently coalesced into marginal strioles; protibia 5–6-spined, with marginal dentation rather well-developed; meso- and metatibia with 4–5 thin, elongate spines, on metatibia mainly along apical half of margin; propygidium with secondary punctures shallow but rather large, uniformly separated by less than half their diameters in basal half, rather discretely obsolete in posterior half; propygidial gland openings present in anterolateral corners but difficult to distinguish from secondary punctures; propygidial strioles absent; pygidium largely lacking secondary punctures; pygidial gland openings not evident; pygidial margin lacking striae. Male (Fig. 25A, H–J): accessory sclerites absent; T8 with ventrolateral apodemes broad, widely separated ventrally; S8 with halves meeting only at basal corner, inner margins short and strongly divergent, apices very narrow, without setae or membranous velum; T9 with apices subacute; T10 deeply, narrowly emarginate at apex; S9 widened to weakly emarginate base, apex narrowly and rather deeply emarginate; tegmen widest just beyond middle, narrowed to apex, apices barely separated, medioventral process produced beneath near midpoint; median lobe about one-third tegmen length, basal piece about half tegmen length.

#### Remarks.

This species is defined by the relatively well developed protibial marginal teeth (Fig. 23D), with deep emarginations between spine insertions. In addition to this character, the species lacks a marginal pygidial stria and secondary pygidial punctures. Its pronotal gland openings are not multiplied, exhibiting only single openings on each side about one-third the pronotal length from the anterior margin, and the sub-basal pygidial gland openings appear to be absent (this is difficult to ascertain). The three specimens attributed here agree in all these characters. However, they also exhibit considerable differences, with the two specimens excluded from the type series showing finer punctation in general, especially more or less lacking elytral interstrial punctures. The Peruvian individual is further distinguished by a rather distinctly explanate pronotal margin. However, as the type is the only male among them, we tentatively lump them together for the present. This will clearly need to be revisited if and when additional material becomes available.

#### Etymology.

This species is named for its distinctively dentate protibiae.

### Crenulister simplex sp. n.

http://zoobank.org/66B616D7-522B-4429-9821-8A956025C163

http://species-id.net/wiki/Crenulister_simplex

Figs 26 –27 , Map 5

#### Type locality.

BOLIVIA: Cochabamba, Valle de Sajta Biological Station [17.1092°S, 64.7978°W].

#### Type material.

**Holotype male:** “BOLIVIA: Cochabamba, Cochabamba, 67.5km NE, Est. Biol. Valle del Sajita[sic], Univ. de San Simon, 300m, 17°6'33"S, 64°47'52"W, 9-13 FEB 1999, R.Hanley, BOL1H99 078, ex. flight intercept trap” / “SM0159345, KUNHM-ENT [barcode label]” (SEMC). **Paratype** (1): **BOLIVIA:** **Santa Cruz**, 4-5km SSE Buena Vista, Hotel Flora y Fauna, 17°29'S, 63°33'W, FIT, 29.iv-6.v.2004, A.R. Cline (LSAM).

#### Diagnostic description.

Length: 1.7–1.8 mm, width: 1.5–1.6 mm; as for generic description with the following diagnostic characters: body rufescent, elongate ovoid, subdepressed; frontal stria fine, complete or narrowly interrupted, frontal disk moderately depressed, with relatively dense punctation consisting of ground punctation and barely larger secondary punctation; epistoma with lateral ridges delimiting median depression bearing weak lateral striae basally; labrum about 4× wider than long, apical margin weakly emarginate; pronotum with gland opening track not quite reaching midline, with 2 openings lying within the impunctate track; pronotal sides weakly explanate, particularly in front; lateral submarginal stria fine, subcarinate, merging with margin just behind anterior corner; pronotal disk with secondary punctures very small and sparse, most evident along basal margin, almost indistinguishable from background punctation elsewhere; weak prescutellar impression present; elytron with one complete, crenulate epipleural stria rather distant from margin, especially posteriorly, both subhumeral striae and dorsal striae 1–4 complete, 5^th^ and sutural striae obsolete in basal third, striae shallowly but coarsely impressed; elytral intervals very sparsely, irregularly punctate, inner intervals with 2–6 very small secondary punctures; prosternal keel deeply emarginate at base, carinal striae slightly abbreviated, divergent anteriorly, not connected; prosternal lobe slightly deflexed, marginal stria present only at middle; mesoventrite with marginal stria fine, not crenulate, mesometaventral stria crenulate, angulately arched forward just short of mesoventral midpoint; postmesocoxal stria recurved anterad around mesocoxa to mesepimeron; lateral metaventral stria rather fine, only weakly crenulate, ending short of metacoxa; metaventral disk with secondary punctures almost obsolete in anterior half and along midline, coarser posterolaterally; metepisternal punctures coalesced into a short stria; lateral stria of 1^st^ abdominal ventrite finely impressed, oblique along inner edge of metacoxa, curving laterad behind metacoxa; secondary punctures of median portion of 1^st^ abdominal ventrite largely restricted to basal third, with slightly oblique punctures toward sides behind metacoxa; punctures along posterior margins of ventrites 1–4 transversely elongate, intermittently coalesced into marginal strioles; protibia ~6-spined, with marginal dentation weakly developed; meso- and metatibia with thin, elongate spines, mainly along apical half of margin; propygidium with secondary punctures shallow but rather large, separated by 1–2× their diameters in basal half, smaller and sparser apically; propygidial gland openings present just mediad lateral corners, nearly one-half behind anterior margin, propygidial strioles absent; pygidium lacking secondary punctation; pygidial gland openings slightly tuberculate, evident near sides about one-fourth from base; pygidial margin with striae along most of apical two-thirds, but interrupted at apex. Male (Fig. 27): accessory sclerites reduced, vestigial; T8 with ventrolateral apodemes slightly narrowed ventrally; S8 with halves meeting only at basal corner, inner margins short and strongly divergent, apices narrow, with three strong setae; T9 with apices subacute; T10 apex entire; S9 widened to bulbous, emarginate base, apex narrowly and deeply emarginate; tegmen widening slightly from base just beyond middle, narrowed to apex, apices narrowly separated, medioventral process produced beneath about one-third from base; median lobe about one-fourth tegmen length, basal piece about one-third tegmen length.

#### Remarks.

**Figure 26.**
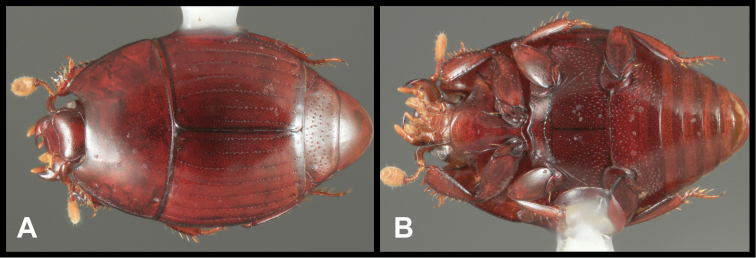
*Crenulister simplex*. **A** Dorsal habitus **B** Ventral habitus.

The basally obsolete 5^th^ and sutural striae (Fig. 26A) in this species are largely adequate to distinguish it. The only other species in the genus with basally weakened striae (*Crenulister explanatus*) has the pronotum and elytral interstriae much more conspicuously punctate. Both are very weakly and finely punctate in this species.

**Figure 27.**
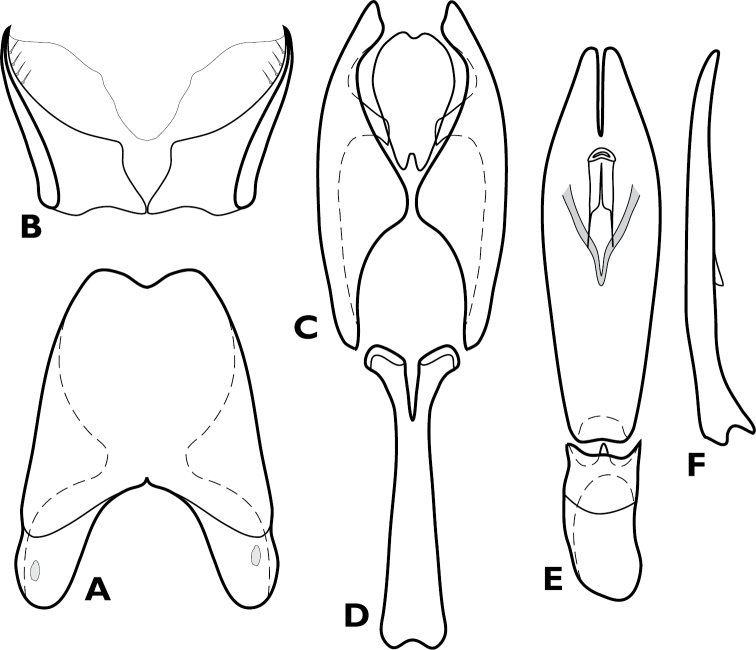
*Crenulister simplex*, male genitalia. **A** 8^th^ tergite **B** 8^th^ sternite **C** 9^th^ and 10^th^ tergites **D** 9^th^ sternite **E** Aedeagus, dorsal view **F** Aedeagus, lateral view.

#### Etymology.

This species’ name refers to its relatively impunctate, simple external sculpturing.

## Supplementary Material

XML Treatment for Conocassis

XML Treatment for Conocassis minor

XML Treatment for Conocassis dromedaria

XML Treatment for Conocassis trisulcata

XML Treatment for Conocassis invaginata

XML Treatment for Enkyosoma

XML Treatment for Enkyosoma rockwelli

XML Treatment for Pluricosta

XML Treatment for Pluricosta onthophiloides

XML Treatment for Pyxister

XML Treatment for Pyxister devorator

XML Treatment for Pyxister labralis

XML Treatment for Chapischema

XML Treatment for Chapischema doppelganger

XML Treatment for Scaptorus

XML Treatment for Scaptorus pyramus

XML Treatment for Lacrimorpha

XML Treatment for Lacrimorpha glabra

XML Treatment for Lacrimorpha subdepressa

XML Treatment for Lacrimorpha balbina

XML Treatment for Lacrimorpha acuminata

XML Treatment for Crenulister

XML Treatment for Crenulister grossus

XML Treatment for Crenulister spinipes

XML Treatment for Crenulister seriatus

XML Treatment for Crenulister paucitans

XML Treatment for Crenulister umbrosus

XML Treatment for Crenulister impar

XML Treatment for Crenulister explanatus

XML Treatment for Crenulister dentatus

XML Treatment for Crenulister simplex

## References

[B1] Caterino MS Tishechkin AK Dégallier N (2012) A revision of the genus *Mecistostethus* Marseul (Coleoptera: Histeridae). ZooKeys 213: 63-78. doi: 10.3897/zookeys.213.3552 PMC3426875 22933855

[B2] Caterino MS Tishechkin AK (2013a) A systematic revision of the genus *Operclipygus* Marseul. ZooKeys 271: 1-401. doi: 10.3897/zookeys.271.4062 PMC3652427 23717185

[B3] Caterino MS Tishechkin AK (2013b) A systematic revision of the genus *Baconia* Lewis. ZooKeys 343: 1-297. doi: 10.3897/zookeys.343.5744 PMC3817432 24194656

[B4] Caterino MS Tishechkin AK (in press) Phylogeny and generic limits in New World Exosternini (Coleoptera: Histeridae: Histerinae). Systematic Entomology.

[B5] Dégallier N Mazur S Tishechkin AK Caterino MS (2012) A revision of the genus *Kaszabister* Mazur. ZooKeys 199: 71-89. doi: 10.3897/zookeys.199.3245 PMC3368282 22711996

[B6] Helava JVT Howden HF Ritchie AJ (1985) A review of the New World genera of the myrmecophilous and termitophilous subfamily Hetaeriinae (Coleoptera: Histeridae). Sociobiology 10(2): 127-382.

[B7] Kanaar P (1997) Revision of the genus *Paratropus* Gerstaecker. Zoologische Verhandelingen 315: 1-185.

[B8] Lawrence JF Ślipiński A Seago AE Thayer MK Newton AF Marvaldi AE (2011) Phylogeny of the Coleoptera based on morphological characters of adults and larvae. Annales Zoologici 61(1): 1-217. doi: 10.3161/000345411X576725

[B9] LeConte JE (1845) A monography of the North American Histeroides. Boston Journal of Natural History 5: 32-86.

[B10] Ôhara M (1994) A revision of the superfamily Histeroidea of Japan (Coleoptera). Insecta Matsumurana, New Series 51: 1-283.

[B11] Wenzel RL Dybas HS (1941) New and little known Neotropical Histeridae (Coleoptera). Fieldiana, Zoology 22(7): 433-472.

